# Road mitigation structures designed for Texas ocelots: Influence of structural characteristics and environmental factors on non-target wildlife usage

**DOI:** 10.1371/journal.pone.0304857

**Published:** 2024-07-22

**Authors:** Anna Rivera Roy, Kevin W. Ryer, Md. Saydur Rahman, John H. Young, Richard J. Kline

**Affiliations:** 1 School of Earth, Environmental and Marine Sciences, University of Texas Rio Grande Valley, Brownsville, Texas, United States of America; 2 School of Integrative Biological and Chemical Sciences, University of Texas Rio Grande Valley, Brownsville, Texas, United States of America; 3 Department of Environmental Affairs, Texas Department of Transportation, Austin, Texas, United States of America; Kerman University of Medical Sciences, ISLAMIC REPUBLIC OF IRAN

## Abstract

Roads negatively impact wildlife through habitat fragmentation, loss of habitat connectivity, and wildlife-vehicle collisions, thus road mitigation structures, such as wildlife crossing structures (WCS), wildlife guards (WG), and fencing are commonly used to address this issue all over the world, including in the United States. In South Texas, such structures were built or modified along a State Highway in an effort to address road mortality for the endangered ocelot (*Leopardus pardalis*) and non-target wildlife species. The goal of this study was to examine temporal changes in wildlife interactions with WCS and WG during and after their construction and modification along a South Texas highway and to determine whether environmental factors influenced use of WCS. Using camera traps deployed to monitor the road mitigation structures, we compared crossing rates, repel rates, and species richness of all species that interacted with the structures, and we examined whether differential wildlife use of WCS and WG was affected by one or more structural dimensions, distance to nearby vegetation, and water presence. Crossings through WCS by wildlife decreased following the completion of construction of mitigation structures; however, repel interactions at WG increased. Overall, crossings decreased at WCS that had higher openness ratios and during periods of precipitation and higher daily temperatures, but distance to vegetation had minimal influence. These factors were shown to influence crossings of each of the five most frequently observed species differently. Lastly, the presence of pooled water at one WCS caused a decrease in crossings when the water level was highest but was not a barrier at lower water levels. By examining influences on wildlife interaction with road mitigation structures, we conclude that a variety of structures, including different WCS configurations, can be beneficial in facilitating movement and restricting entry into the right-of-way for a diversity of wildlife species beyond the target species.

## Introduction

Roads are known to influence wildlife distributions, movements, and survival [1]. Roads are a direct contributor to habitat loss and fragmentation, thus creating and isolating smaller populations by limiting dispersal [1–3]. Measures to reduce the negative effects of roads on wildlife often include road mitigation structures, which have been developed to both restrict wildlife from entering roadways, through structures such as wildlife guards (WG) and fencing, and safely facilitate their movements across roads, through wildlife crossing structures (WCS) [4–6]. The effectiveness of these structures is often determined by whether they improve connectivity between habitat areas, whether they reduce road mortalities, or both [4].

One way to begin assessing the effectiveness of road mitigation structures is to examine how wildlife interact with them over time. Dodd et al. [7], Braden et al. [8], and Alonso et al. [9] reported an increase in WCS use from pre-construction to post-construction of mitigation structures when assessing WCS use in Florida [7, 8] and southern California [9]. Importantly, McCollister and van Manen [10] and Seidler et al. [11] documented that the construction of fencing and WCS contributed to a decrease in wildlife road mortalities along US 64 in Washington County, North Carolina and pronghorn mortalities along US 191 in western Wyoming, respectively. Increases in wildlife use of WCS have led to a supposition that wildlife becomes more comfortable with WCS over time, demonstrating one way that WCS can be effective in conveying movement where roads disrupt habitat connectivity [8, 9, 12].

Belant et al. [13] and Sebesta et al. [14] found in their respective studies on white-tailed deer (*Odocoileus virginianus*) in Erie County, Ohio and San Patricio County, Texas that cattle and deer guards with straight crossbars were effective at restricting ungulate movement. Moreover, Peterson et al. [15] and Allen et al. [16] demonstrated that deer and WG with crossed or bridge grating configurations were also effective for ungulates along US 1 in the Florida Keys and US 93 in northwestern Montana. While WG were not completely effective at deterring black bear (*Ursus americanus*) and coyote (*Canis latrans*), Allen et al. [16] found that when WG were paired with WCS, all black bear and 94.7% of coyotes used WCS to cross the road.

Setting up a study with a before-after-control-impact (BACI) to monitor areas before and after mitigation and at sites unaffected by human impact (control) and those that are (impact) has been described as the optimal study design to measure mitigation structure effectiveness [17–19]. When a full BACI study is not possible, a before-after (BA) study may take its place [17–19]. Several studies have documented changes in wildlife use of crossing structures from pre-construction of road mitigation to post-construction [7–10]; however, few studies have examined the use of mitigation structures during construction [11, 20].

Monitoring the number of successful crossings by wildlife is insufficient for evaluating the effectiveness of crossing structures [18, 19]. Use of crossing rates and performance ratios using the minimum expected rate of use supplies a better comparison of the potential for wildlife to use WCS and the actual use of WCS [18, 19, 21]. Control plots are typically used with crossing-use measurements to calculate the minimum expected rate of use [19]; however, when control plots are not available, crossing rates have been used to measure the performance of WCS [22].

In examining wildlife use of WCS, it is important not only to look at the frequency of use and the differential use of WCS by multiple species but also to find possible reasons why these differences are being observed. Variables in the form of structural characteristics and environmental factors are important in attempting to explain differences in crossing structure usage [18, 19, 21, 23]. Previous studies have considered dimension [23–25], presence of water [26], noxious noise [21, 23], human activity [21, 23], and nearby vegetation [27, 28] as important factors that may influence the effectiveness of WCS. Studies have linked differential species’ use of underpasses to the openness ratio, which is the calculation of (width × height) ÷ length [21, 23, 29], as well as the presence of flowing water [26] and vegetative cover [27]. Importantly, understanding the relationship between the effectiveness of mitigation measures and their structural characteristics and environmental factors can aid in informing the optimal placement and design of future mitigation structures [4, 30].

In many instances, WCS placement is, or should be, based on locations of high-quality habitat fragments to increase connectivity between them [9, 31]. As such, some factors such as distance to vegetation or natural habitat and daily precipitation may also be important factors affecting WCS use. Some species, such as ocelots, require dense vegetation [32] and likely certain proximity of vegetation to WCS entrances. Additionally, heavy precipitation events occasionally creates flooded conditions which may reduce WCS use. Episodic inundation occurred at one WCS along with Texas SH 100 due to heavy rainfall and provided a unique opportunity to examine how water presence influences WCS use [33]. An understanding of how and why wildlife differentially use WCS based on structural characteristics will better inform wildlife management in this unique system that supports endangered species.

Newly constructed and modified WCS, WG, and exclusionary fencing allow for an opportunity to examine how wildlife interactions with these structures change over time. WCS of various sizes and designs were constructed by the Texas Department of Transportation (TxDOT) on State Highway (SH) 100 located in Cameron County in South Texas to reduce ocelot (*Leopardus pardalis albescens*, an endangered species) road mortality and improve connectivity among wildlife populations [34]. The placement of the WCS was based on previous construction of concrete traffic barriers that hindered ocelot movement across the highway and resulted in three ocelot mortalities [34]. Despite the fact that the WCS were intended for ocelot use, the structures have facilitated movement of many non-target species. Moreover, WCS along SH 100 provides an opportunity to examine how different characteristics of WCS influence their use by both target and non-target species. Overall, use (completed passage) is the desired behavior at WCS, and non-use (repel) is the desired behavior at WG [22]. Understanding the frequency of these behaviors at mitigation structures will aid in determining their effectiveness in facilitating wildlife movement and potentially reducing wildlife road mortalities.

The objectives of this study were to examine how both target and non-target species use of mitigation structures changes from during construction to post-construction periods and to examine how structural and environmental conditions may affect use. We expected that species richness observed from wildlife cameras would increase at mitigation structures from during construction to the post-construction periods on State Highway 100 (SH 100). We hypothesized that successful crossing rates at WCS would increase and repel rates would increase at wildlife guards (WG) in the post-construction period. Further, during periods of flooding at the WCS, we hypothesized that observations and crossing rates of wildlife would decrease.

## Materials and methods

### Study area

**Fig 1:**
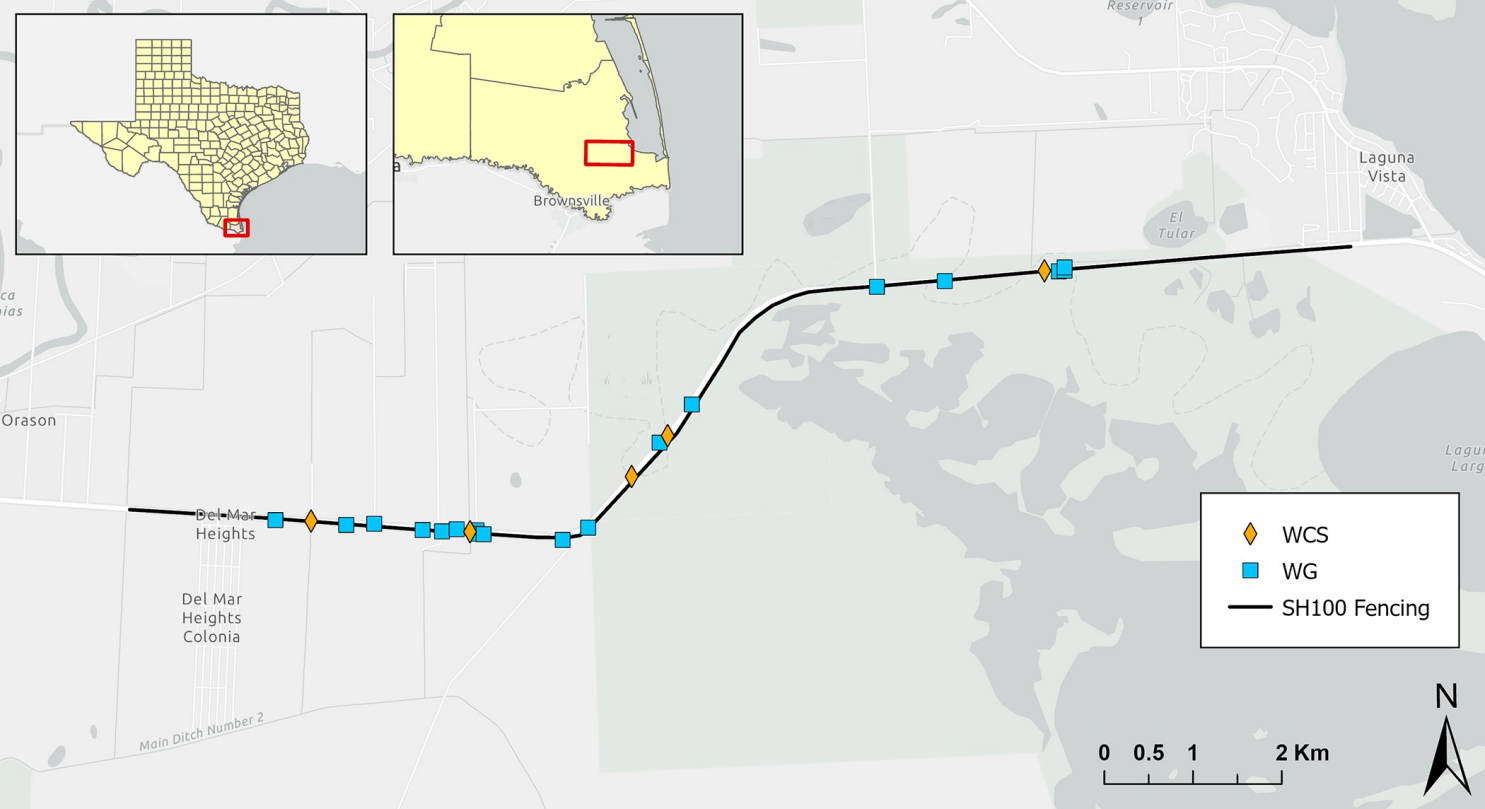
Wildlife crossing structures (WCS; n = 5), 11.9 km of fencing, and wildlife guards (WG; n = 18) constructed by the Texas Department of Transportation to address ocelots and other animals’ mortality along State Highway 100 in Cameron County, Texas, USA. All structures were monitored using remote cameras. Data were collected at WCS from January 2017 to May 2019 and at WG from April 2017 to May 2019. Basemap provided by Esri. Esri reserves the right to grant permission for any other use of the Image.

This study was conducted along an 11.9 km stretch of SH 100 between Los Fresnos and Laguna Vista in Cameron County, Texas (Fig 1). The road is flanked by the U.S. Fish and Wildlife Service (USFWS) protected Laguna Atascosa National Wildlife Refuge (LANWR) and agricultural and residential land. The study area is located within the Rio Grande Delta physiographic zone [35]. The surrounding habitat is characterized by Texas coastal grassland, Tamaulipan saline thornscrub, and Texas coastal high salt marsh [35, 36]. The diversity of ecological systems and land use along with SH 100 result in varying amounts of vegetative cover, from open sea-ox-eye daisy (*Borrichia frutescens*) flats to smooth cordgrass (*Spartina alterniflora*) fields, to thornscrub patches with canopy cover [35, 37]. The area receives an average annual rainfall of 66 cm, with highs in the summer reaching an average temperature of 33°C and lows in the winter getting down to an average of 12°C [38].

**Fig 2:**
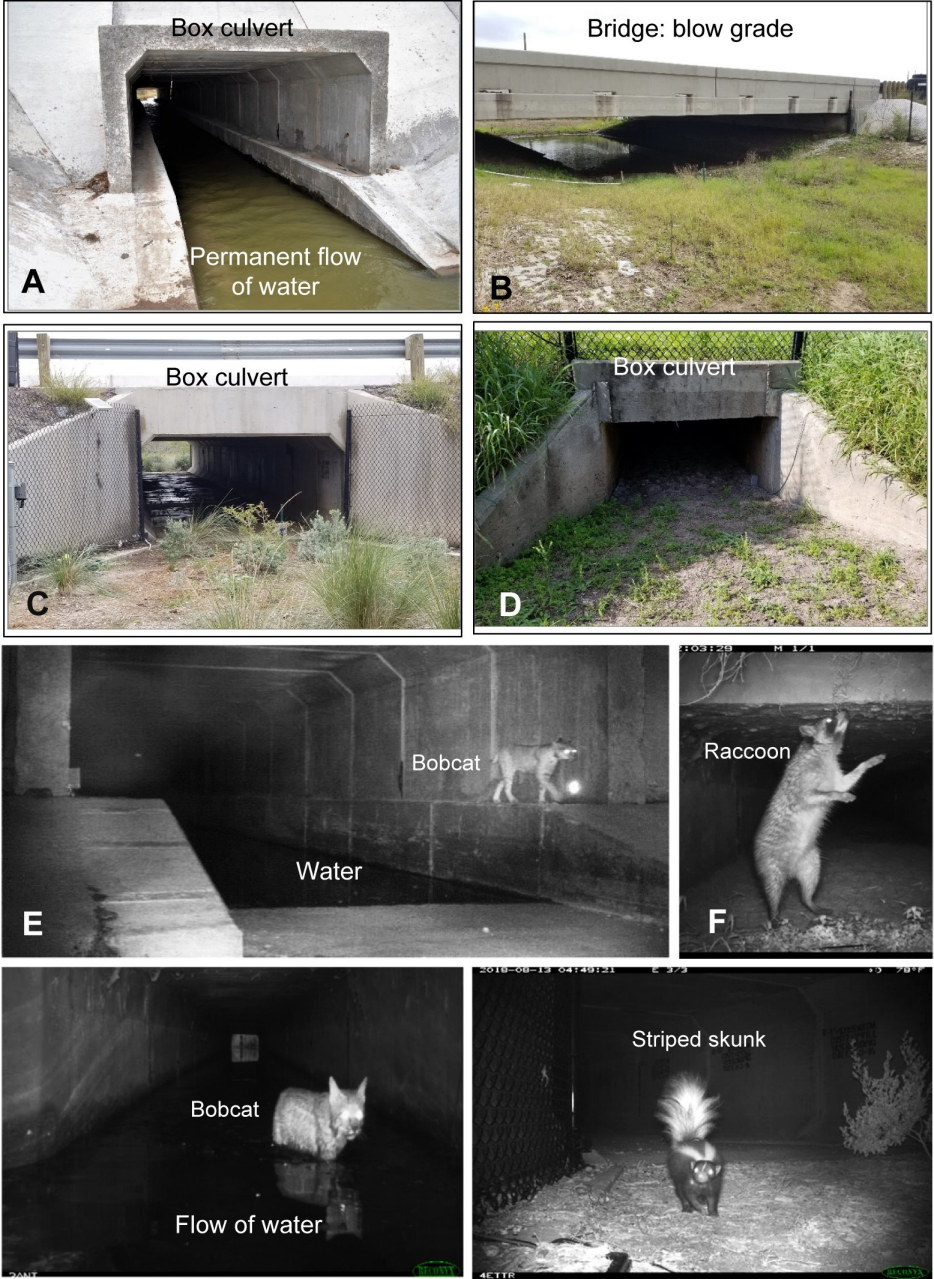
Four of the five wildlife crossing structures (WCS) constructed by the Texas Department of Transportation to address ocelot (*Leopardus pardalis albescens*) mortality along State Highway 100 in Cameron County, Texas. (A) Box culvert, openness ratios: 04 and 0.36 (WCS2); (B) Bridge: below grade, openness ratio: 1.76 (WCS3); (C) Box culvert: below grade, openness ratio: 0.21 (WCS4); (D) Box culvert: above grade, openness ratio: 0.62 (WCS3A). Note: WCS1 is not pictured because it has the same configuration as WCS2. (E-H) Examples of pictures from the camera showing wildlife crossing WCS at night.

**Fig 3:**
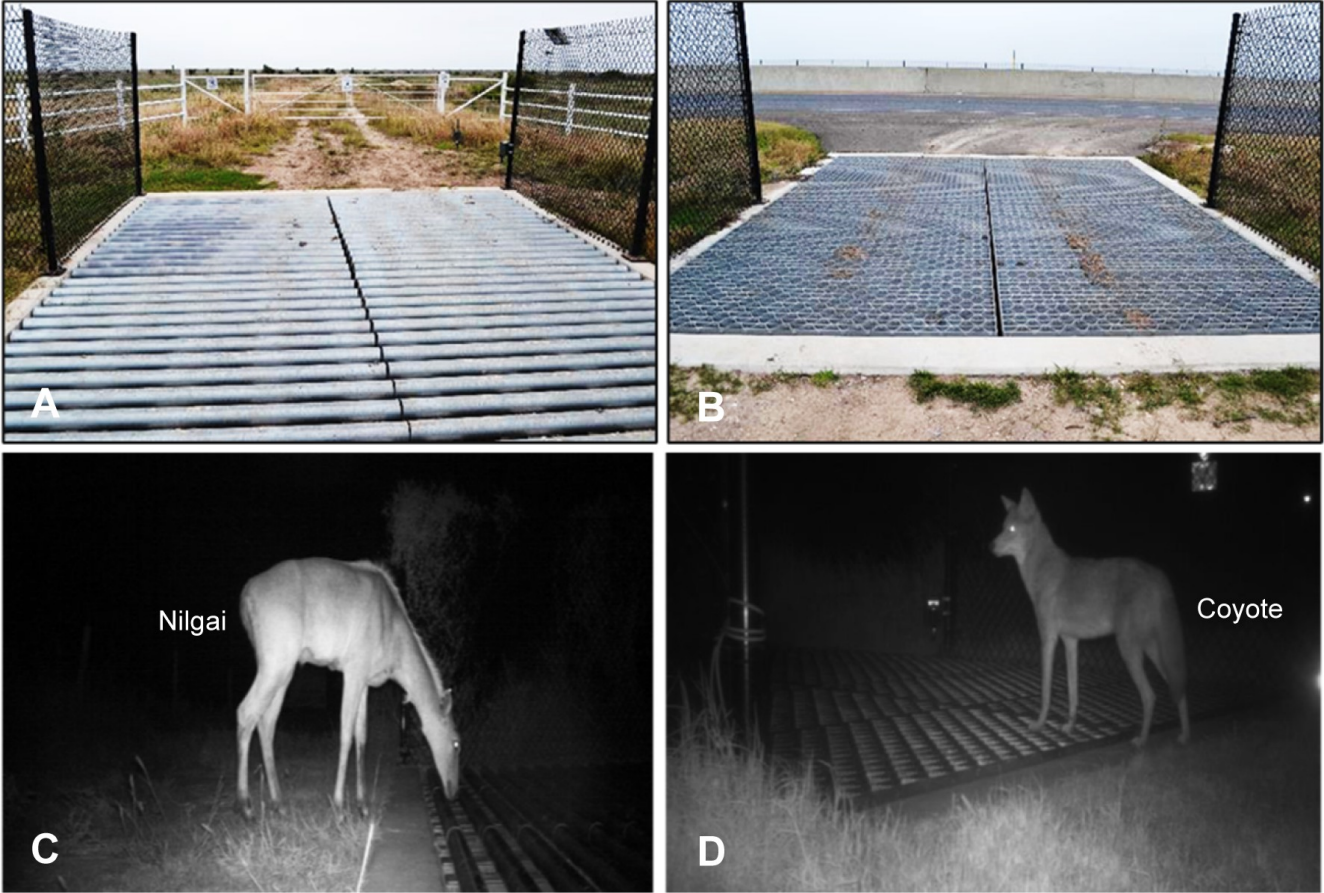
Two wildlife guard (WG) types were constructed by the Texas Department of Transportation to address ocelot mortality along State Highway 100 in Cameron County, Texas; (A) pipe and (B) bridge grating. (C, D) Examples of pictures from the camera showing wildlife at WG at night.

Construction of road mitigation structures resulted in a 1.8 m tall chain-link fence spanning an 11.9 km length of SH 100, on the north and south sides of the highway, along with five WCS (Fig 2) and 18 WG (Fig 3). WCS1 and WCS2 were large concrete box culverts, each located in 3- and 5-meter-deep drainage ditches with permanent water respectively. A concrete walkway was installed on both sides above the water line to enable wildlife passage (S1 Table). WCS3 was a newly constructed bridge underpass with a natural substrate and ephemeral water presence (S1 Table). WCS3A was a small concrete box culvert with a dirt substrate and ephemeral water presence (S1 Table). WCS3A was already in existence prior to the construction of the other WCS along SH 100 and was only modified during the construction period with fence replacement and cutting back vegetation. Lastly, WCS4 was a newly constructed medium concrete box culvert with a concrete substrate and no water presence (S1 Table). Construction began in September 2016 and was completed in May 2018.

### Data collection

**Fig 4:**
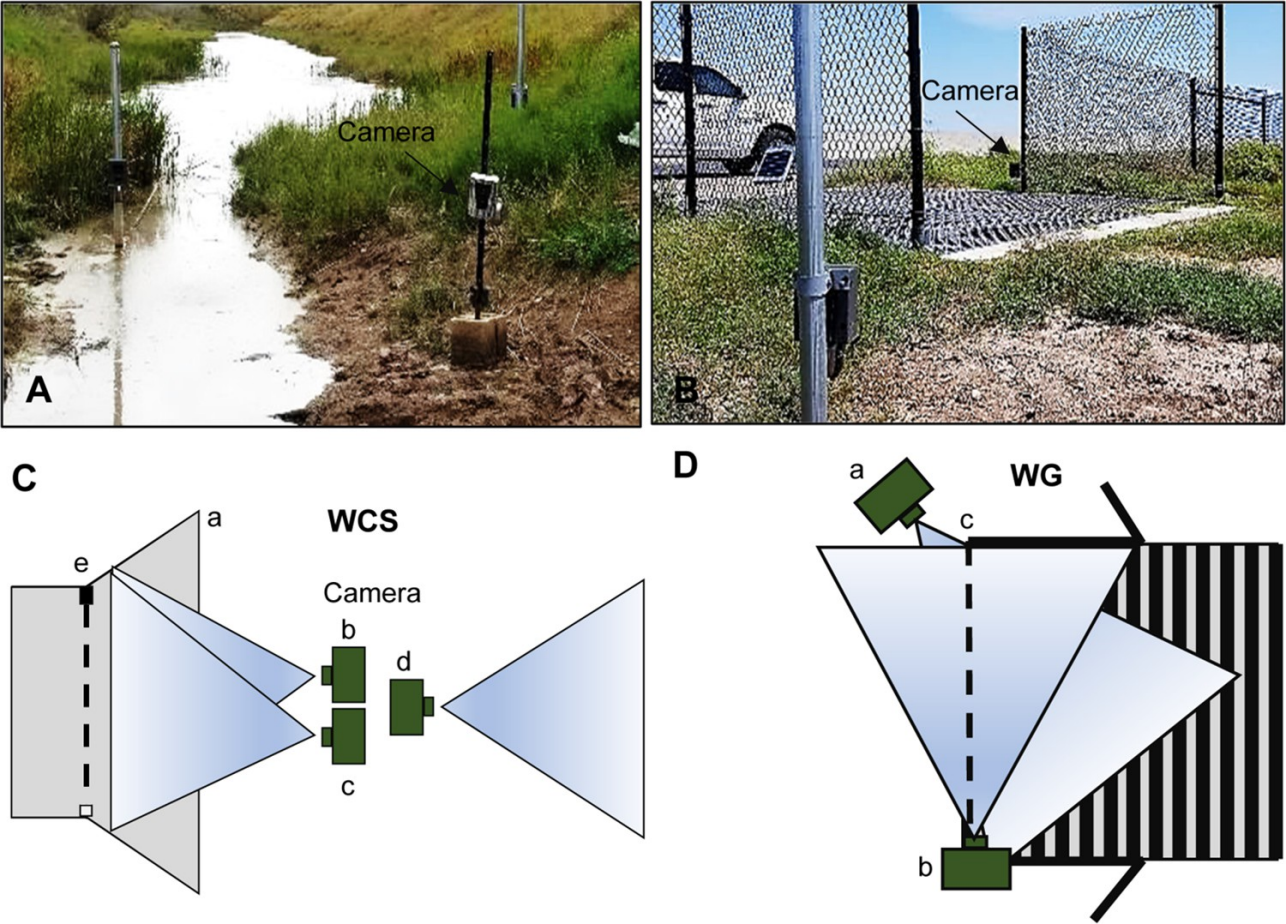
Example of camera trap setup. Cameras were placed at the opening of a wildlife crossing structures (WCS; A) and wildlife guards (WG; B) constructed by the Texas Department of Transportation to protect ocelots and other animals along State Highway 100 in Cameron County in Texas from January 2017 to May 2019. (C) Schematic diagram of an active infrared camera (b) and one video camera (c) facing toward the WCS opening (a) while one passive infrared camera (d) faces away. The external sensing system is represented by the black and white boxes connected with the dashed line (e). (D) Schematic diagram of an active infrared camera (a) facing toward the WG and road while one passive infrared camera (b) faces toward the habitat side. The external sensing system is represented by the dashed line (c).

Species activity at mitigation structures was monitored using active infrared (AIR) and passive infrared (PIR) triggered camera traps (Reconyx PC900 HyperFire™ and Reconyx HS2X HyperFire 2™; Reconyx LLP, Holmen, WI, USA) placed at each WCS and WG. Monitoring occurred from January 2017 to May 2019 at WCS and from April 2017 to May 2019 at WG. Each WCS had two cameras on each side of the WCS, with one camera trap facing toward the opening and one facing away (Fig 4). One camera was equipped with both PIR and AIR triggers to capture wildlife interacting with the WCS. A second PIR-only camera trap faced away from the WCS to capture wildlife in the immediate surrounding areas that may not interact with the WCS (Fig 4). The PIR trigger is activated within the camera by changes in radiation emitted in the camera’s field of view, while the AIR trigger is activated by an externally triggered infrared beam deployed in the field of view [39]. Additionally, video cameras (Bushnell Trophy Cam HD Model 119874, Bushnell Corporation, Overland Park, KS, USA, and Reconyx HyperFire 2™) were set up at each WCS entrance to supplement still photographs in the wildlife-WCS interaction analysis. Additional cameras were set up at larger WCS openings to ensure that the full extent of the opening was captured and that the number of individual animals missed by the cameras was reduced. Each of the 16 monitored WG were equipped with one AIR-equipped camera facing the WG and road and one PIR-only camera facing away from the WG toward the habitat (Fig 4). Each camera in this array was checked every two weeks to exchange memory cards for empty ones, change batteries as needed, and check for any maintenance issues. This research was conducted within the defined right-of-way of SH 100; therefore, no permits were necessary.

To determine how WCS characteristics influence wildlife use, structural characteristics, and environmental factors for each WCS along SH 100 were compiled. Wildlife crossing structure dimensions (length, width, and height) and openness ratio were previously obtained by TxDOT [34]. To determine the influence of vegetation on WCS use, the distance to the nearest patch of native vegetation from the WCS entrances was measured for WCS3, WCS3A, and WCS4 using a measuring tape. To calculate an average distance to vegetation at each crossing opening, three distance measurements were taken at each WCS opening: two following the lateral fence edges from the opening to vegetation and one from the center of the opening directly to the vegetation. Because WCS1 and WCS2 were situated in drainage ditches, the distance from the WCS opening to the top of the ditch and from the top of the ditch to the nearest large patch of native vegetation was measured. Finally, daily precipitation and daily low temperatures were obtained from NOAA Climate Data Online [38].

**Fig 5:**
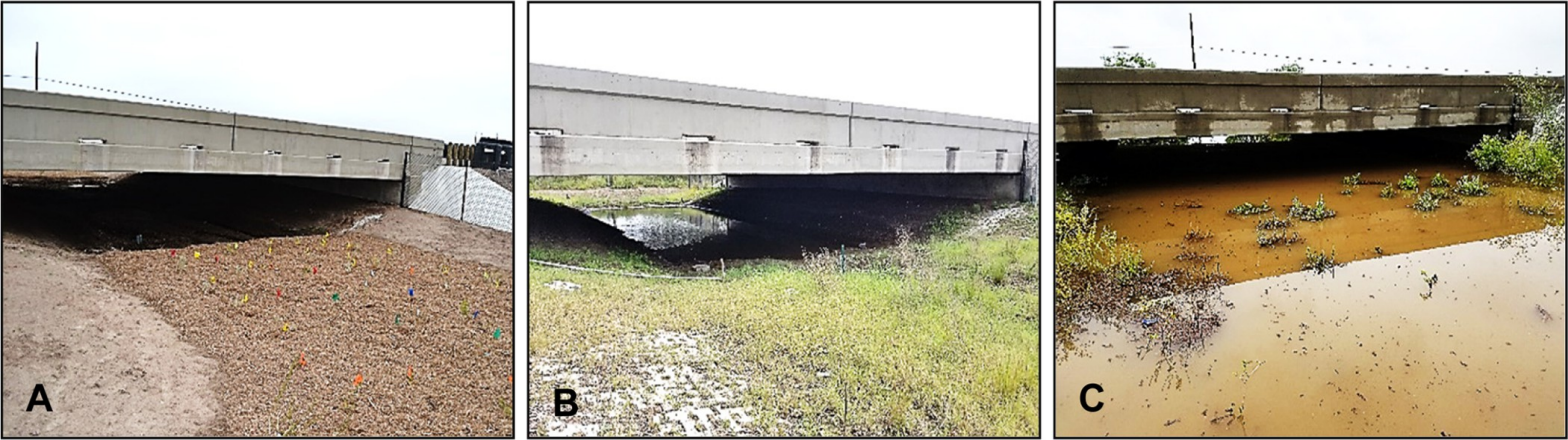
Water inundation at one wildlife crossing structure (bridge: Below grade), located on State Highway 100 in Cameron County, Texas, occurring between January 2018 and May 2019. Water levels depicted: little to no water (A); intermediate pooling of water (B); full of water (C). Reprinted from [40] under a CC BY license, with permission from A. D. Rivera Roy, original copyright 2020.

To understand how standing water influenced WCS use, the amount of water at WCS3 was categorized for comparison against wildlife crossing rates. At this WCS, water was pumped intermittently to remove pooled water using a gas-powered pump and hose (Model 100113, Champion Power Equipment, Santa Fe Springs, CA, USA). The level of water pooling was separated into three categories: “0,” with very little to no water pooled under the WCS; “1,” with some water pooled and enough dry pathway for wildlife to cross under the WCS; “2,” with water pooled at its highest level and no room for wildlife to cross under the WCS (Fig 5). The influence of water inundation at WCS1 and WCS2 was not analyzed due to the submersion and damage of monitoring equipment that resulted in a lack of data.

### Data management

To process and organize all photographs and videos, a suite of software programs developed by Sanderson and Harris [41] was used. Each picture was relabeled with its timestamp using the programs Renamer and special Renamer. The pictures from each location were then sorted into file folders based on species, then sorted again by the number of individuals observed in each picture. The program DataOrganize was used to process the sorted file folder structure to catalog all pictures.

### Data analysis

**Fig 6:**
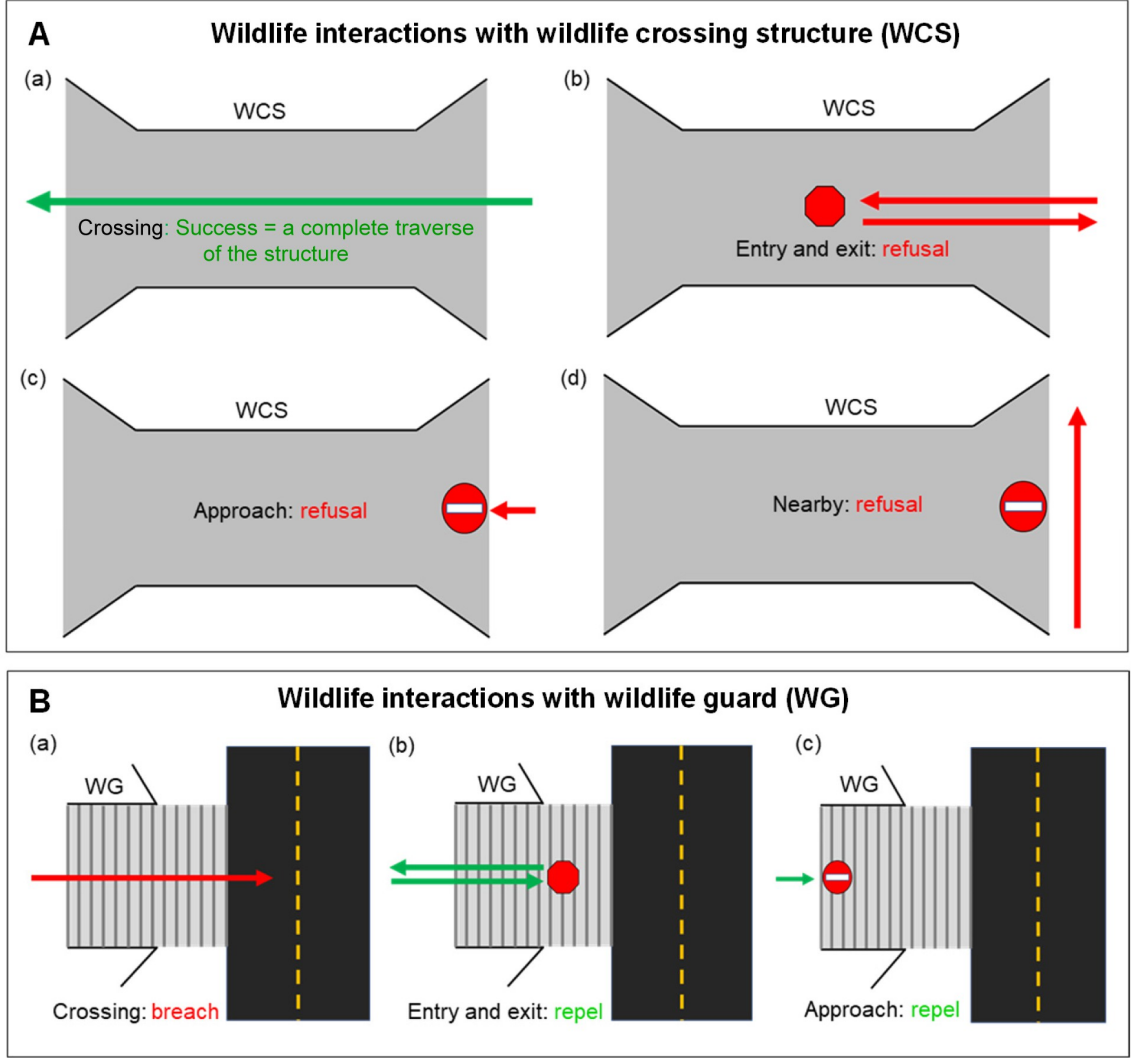
Schematic diagram of wildlife interactions with wildlife crossing structures (WCS) and wildlife guards (WG). (A) WCS and (B) WG.

To determine how wildlife interacted with WCS, behaviors were categorized into four different groups: (1) “crossing,” in which individuals completely cross from one end of the WCS to the other; (2) “entry” and exit on the same side, where individuals do not complete movement to the opposite end; (3) “approach” without entry; and (4) “nearby,” where individuals are in the vicinity of a WCS but do not interact with it (Fig 6A). Crossing, entry, approach, and nearby were used to calculate a crossing rate for WCS using the following formula:

$$Crossing\;rate=\frac{\sum no.of\;crossings}{\sum \left(no.of\;entry+no.of\;approach+no.of\;nearby\right)}$$

Crossing, entry, and approach were used to calculate a repel rate for WG using the following formula:

$$repel\;rate=\;\frac{\sum \left(no.of\;entry+no.of\;approach\right)}{(\sum no.of\;crossing+no.of\;entry+no.of\;approach)}$$

Nearby interactions were not included in WG repel rates as wildlife may have been traveling parallel to the fence without apparent intention to enter the roadway where they are meant to be deterred. Directional movements were also recorded at WG and categorized into habitat-road and road-habitat. Only habitat-road interactions were included in repel rates because entry and approach behaviors could not be observed from the roadside of WG (Fig 6B).

Monitoring was broken into two time periods: during construction (January 2017 –May 2018 at WCS; April 2017 –May 2018 at WG) and post-construction (May 2018 –May 2019). The construction of WG began later than when construction of WCS was initiated. When cameras were occasionally temporarily removed from sites due to flooding or repair, interactions with mitigation structures could not be observed. Only data from dates, when at least one AIR-equipped camera was active at a WCS were used in data analysis. Additionally, only data from dates when the AIR-equipped camera was functioning were included in the analysis. Only photographs of identifiable mammal species were used in the analysis of WCS and WG (S2 Table); birds, rodents, and unidentifiable individuals were excluded. All herpetofauna that were not of conservation concern were grouped together. Reptile species of conservation concern consisted of the Texas tortoise (*Gopherus berlandieri*) and Texas indigo snake (*Drymarchon melanurus erebennus*) documented as individual species.

Data were binned by monthly increments to serve as replicates. For comparing WCS uses during and post-construction, only WCS1, WCS2, and WCS3A were included in the analysis. Monitoring at newly constructed WCS3 and WCS4 did not begin until post-construction due to incomplete construction of surrounding fencing until that time and were not included in the analysis, though species use of these two WCS was recorded (S4 Table). All means are reported as mean ± standard error. To gain a basic understanding of whether daily wildlife activity was different between during and post-construction periods and between WCS, a permutational analysis of variance (PERMANOVA) was performed using daily counts of crossings, and another PERMANOVA was performed using daily counts of refusals.

To determine whether there was an increase in crossing rates between the during and post-construction periods, a two-way analysis of variance (ANOVA) was conducted using program R 3.6.1 comparing monthly crossing rates to site and construction periods [42]. Crossing rates were arranged into a matrix and imported into PRIMER v7 (Primer-e, Ltd. Plymouth Marine Laboratory, UK) for analysis. Monthly bins by location served as samples, and species and species groups served as variables. A Bray-Curtis similarity matrix was calculated in PRIMER with untransformed data and a dummy variable of one as a zero adjustment [43]. Then, a permutated analysis of variance (PERMANOVA) was used to compare crossing rates of wildlife communities between construction periods nested within WCS. A post-hoc pairwise PERMANOVA was used to compare crossing rates of communities between during and post-construction periods for each individual WCS. A test for homogeneity of dispersion (PERMDISP) was performed in PRIMER to test the dispersion of samples across WCS and the construction period [44]. Using a similarly constructed matrix to that which was used for PERMANOVA, with monthly counts of crossings for each species and species group, a similarity percentages (SIMPER) analysis was performed in PRIMER to determine which species contributed most to similarities of communities crossing observed within the during and post-construction periods for WCS1, WCS2, and WCS3A [43]. Monthly species richness, defined as the count of the number of species present [45], was calculated in PRIMER for species crossing in each construction period at each WCS. A two-way ANOVA was performed to compare species richness between WCS and the construction period using R, and a post-hoc Tukey test (*P*<0.05) was performed for pairwise comparisons.

To gain an understanding of whether daily wildlife activity was different between during and post-construction periods and between pipe wildlife guards (PWG) and bridge grating wildlife guards (BGWG), a PERMANOVA was performed using daily counts of repels, and another PERMANOVA was performed using daily counts of crossings. To determine whether there was an increase in repel rates at WG between the during and post-construction periods, a Wilcoxon rank-sum test was conducted comparing monthly repel rates between the two periods using R [42]. Repel rates were entered into a matrix in PRIMER. Species and species groups served as variables, and monthly data bins by WG type and construction period served as samples. A Bray-Curtis similarity matrix was then calculated with untransformed data and a dummy variable of one in PRIMER for subsequent analyses [43]. Using PERMANOVA, wildlife community repel rates were compared between the during and post-construction periods nested within the WG type. A post-hoc pairwise PERMANOVA was then used to determine whether there was a difference in community repel rates between the during and post-construction periods within each WG type. A test for homogeneity of dispersion (PERMDISP) was performed in PRIMER to test the dispersion of samples across WG type and construction period [44]. Using counts of repels, a SIMPER analysis was used in PRIMER to determine the amount to which each species contributed to similarities within communities being repelled by each WG and each construction period. Monthly species richness was calculated for each WG type for each construction period in PRIMER for species that were repelled by WG. A two-way ANOVA was used to compare differences in species richness between each construction period and each WG type.

To find potential relationships between structural, environmental factors and full crossings of wildlife at all five WCS, post-construction, a generalized linear model (GLM) with a Poisson error distribution was performed using the *stats* package in Program R 3.6.1 [42]. The global model included openness ratio (0.06–0.54 m), height (1.2–2.1 m), length (22.6–54.9 m), width (1.8–6.1 m), daily precipitation (0–11.9 cm), daily low temperature (2.2–27.2°C), daily high temperature (3.9–38.9°C), and distance from WCS entrance to nearest large vegetation patch (0–83.9 m) as factors (S3 Table), and daily counts of successful crossings of all species were the dependent variable.

Individual species models were used to find relationships between the factors tested in the global model and counts of successful crossings of the five most frequently observed species: coyote (*Canis latrans*), bobcat (*Lynx rufus*), raccoon (*Procyon lotor*), opossum (*Didelphis virginianus*), and nine-banded armadillo (*Dasypus novemcinctus*). Because daily counts of crossings for each species were low, often “1” or “0,” individual species counts of crossings were converted to binomial notation, and a binomial distribution was used for each species model. To determine which factors to include in each of these models, the dredge function from the MuMIn package was used as a model-selecting tool based on the lowest change in the Akaike information criterion, or ΔAICc [44, 46].

Differences in wildlife crossing rates for all species combined at the three assigned water levels at WCS3 were tested with a Kruskal-Wallis’s test, followed by a post-hoc Dunn’s test with a Bonferroni correction to discern differences between pairs of water levels. These calculations were performed in Program R; Dunn’s test was performed using the FSA package [47].

## Results

### WCS and wildlife interactions

**Fig 7:**
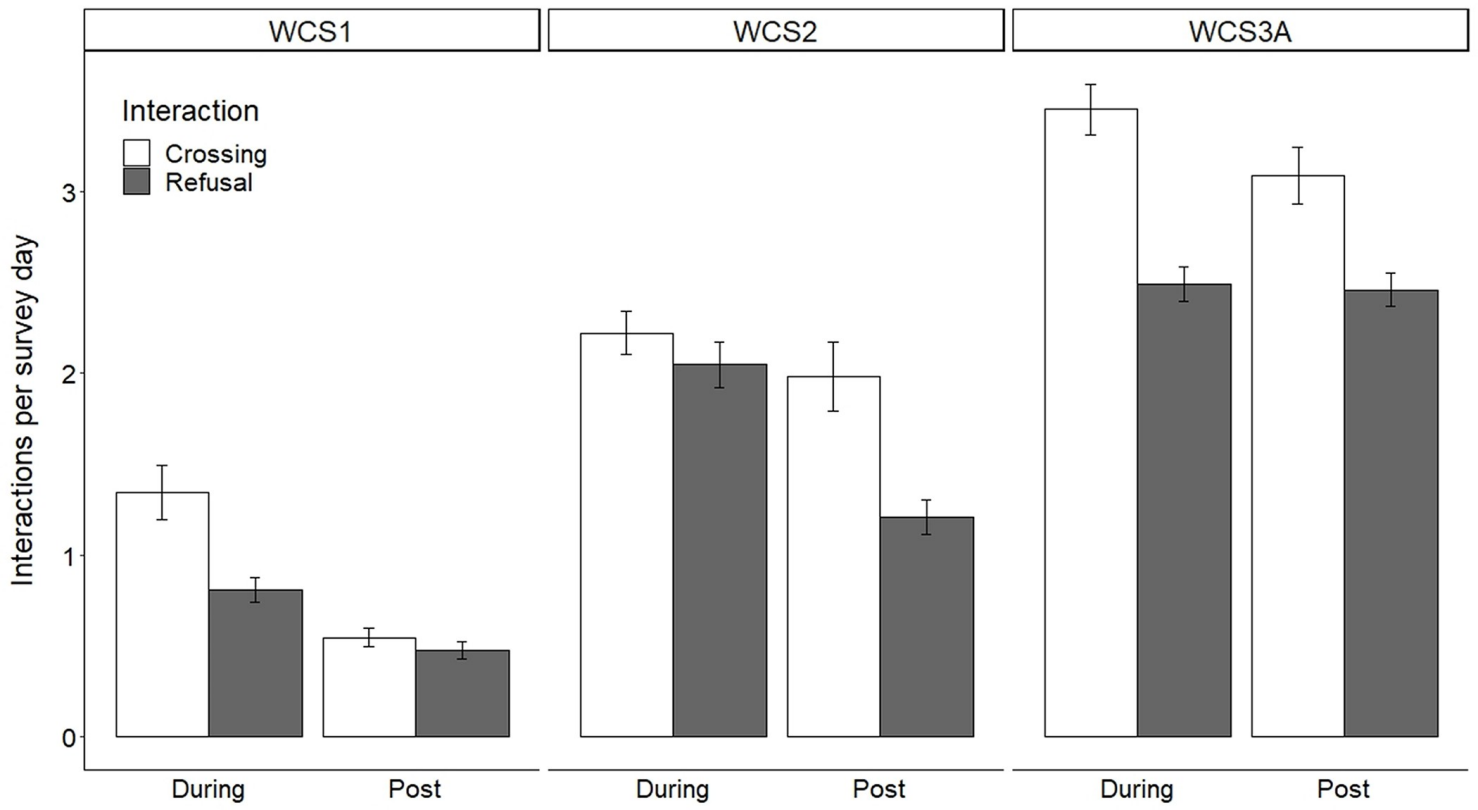
Bar graph showing mean number of crossings and refusals per survey day ± standard error at each wildlife crossing structure (WCS) during construction (January 2017—May 2018) and post construction (May 2018—May 2019). Crossings and refusals were significantly different between WCS1, WCS2, and WCS3A (crossings: *P* = 0.001; refusals: *P* = 0.001) and significantly higher during construction than post construction (crossings: *P* = 0.001; refusals: *P* = 0.001) along State Highway 100 in Cameron County, Texas.

Cameras were deployed for 1,078 survey days during construction and 1,019 survey days post-construction at WCS1, WCS2, and WCS3A combined (Table 1). At WCS1, WCS2, and WCS3A, 4,512 interactions were recorded during construction, and 3,369 interactions were recorded post-construction (Table 2). During construction, WCS1 had a mean of 1.34±0.15 crossings and 0.81±0.07 refusals occurred per survey day; post-construction, a mean of 0.54±0.05 crossings and 0.48±0.05 refusals per survey day were observed (Fig 7). At WCS2 during construction, a mean of 2.22±0.12 crossings and a mean of 2.05±0.13 refusals occurred per survey day. A mean of 1.98±0.19 crossings and a mean of 1.21±0.09 refusals per survey day were observed post-construction. During construction at WCS3A, there were 3.45±0.14 crossings and 2.49±0.09 refusals occurred per survey day, and a mean of 3.09±0.16 crossings and 2.46±0.09 refusals occurred per survey day post-construction. A PERMANOVA showed that the mean daily number of crossings per survey day was significantly different between WCS1, WCS2, and WCS3A (*Pseudo-F*_2_ = 184.6, *P* = 0.001) and between during construction and post-construction periods (*Pseudo-F*_1_ = 62.48 *P* = 0.001). The interaction between the site and the construction period was also significant (*Pseudo-F*_2_ = 6.05, *P* = 0.002). A PERMANOVA showed that the mean daily number of refusals per survey day was significantly different between WCS1, WCS2, and WCS3A (*Pseudo-F*_2_ = 251.3, *P* = 0.001) and between during construction and post-construction (*Pseudo-F*_1_ = 26.3, *P* = 0.001). The interaction between site and construction period for mean daily refusals was significant (*Pseudo-F*_2_ = 13.3, *P* = 0.001) (Fig 7).

**Table 1:** Most abundant species crossing at each wildlife crossing structure (WCS), during and post construction periods, based on similarity percentage analysis of data collected from January 2017 to May 2019 in Cameron County, Texas. Average number of individuals denotes average number of individuals crossing per month, and similarity percentage refers to the extent to which each species contributed to the similarities of communities observed crossing at each WCS and time period.

WCS	During			Post		
	Species	Average no. individuals	Similarity percentage	Species	Average no. individuals	Similarity percentage
1	Raccoon	35.1	99.9	Raccoon	11.7	91.8
				Bobcat	1.2	4.4
				Opossum	0.7	3.8
2	Raccoon	58.6	99.9	Raccoon	10.9	55.6
				Opossum	36.2	35.6
				Bobcat	2.6	8.8
3A	Opossum	56.8	65.3	Opossum	42.9	54.6
	Coyote	14.2	12.5	Bobcat	10.0	12.2
	Raccoon	6.3	10.2	Nine-banded armadillo	9.5	11.3
	Nine-banded armadillo	8.2	9.7	Coyote	8.2	7.7
	Bobcat	2.1	2.1	Raccoon	4.1	7.6
				Javelina	7.5	4.5
				Texas indigo snake	0.8	0.9

**Table 2:** Number of wildlife interactions determined by photo evaluation and count of different species detected by cameras at three wildlife crossing structures (WCS) constructed by the Texas Department of Transportation to protect ocelots and other animals’ motility on State Highway 100 in Cameron County, Texas. Interactions observed at these three WCS were compared during construction versus post construction. Other WCS were excluded from this as they were still being built during the construction period. Monitoring was broken into two-time periods; during construction (January 2017—May 2018) and post construction (May 2018—May 2019).

WCS		Interactions					Species Count			
	During			Post			During		Post	
	Crossing	Refusal	Total	Crossing	Refusal	Total	Crossing	Total	Crossing	Total
1	394	236	630	182	159	341	4	9	6	11
2	651	600	1251	648	395	1043	3	12	5	15
3A	1526	1105	2631	1108	877	1985	11	17	11	18

**Fig 8:**
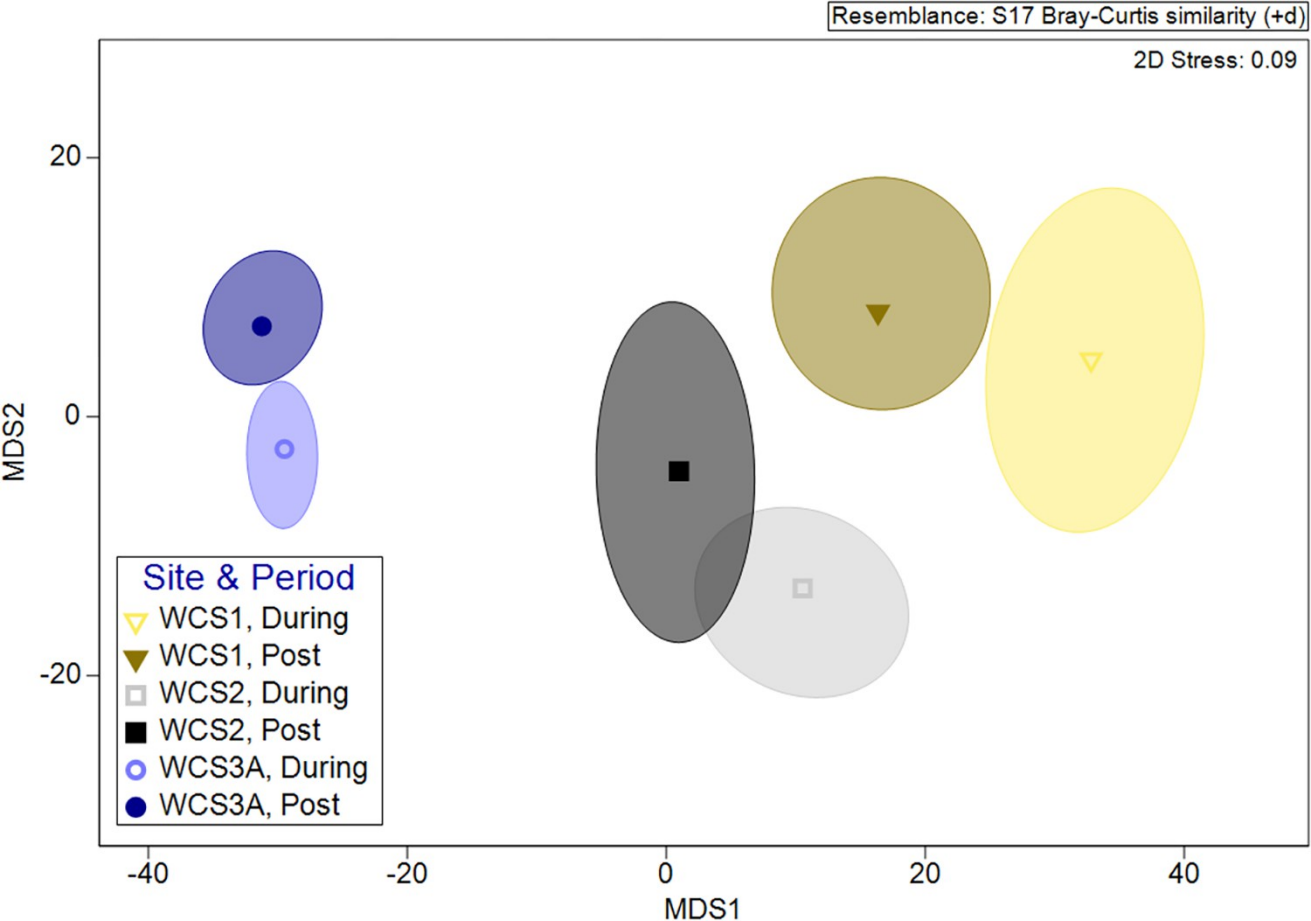
Bootstrapped metric multidimensional scaling (MDS) plot showing differences in monthly crossing rates between communities observed at three wildlife crossing structures (*P≤*0.001) and between during construction (Jan 2017-May 2018) and post construction (May 2018-May 2019; *P≤*0.001) along State Highway 100 in Cameron County, Texas. Differences are indicated by physical distance between bootstrapped averages surrounded by 95% confidence areas.

The mean monthly crossing rate for all species at WCS1 during construction was 0.59±0.04, and the mean monthly crossing rate post-construction was 0.51±0.05 (S1 Fig). For all species at WCS2, the mean monthly crossing rate during construction was 0.52±0.04, and the mean monthly crossing rate post-construction was 0.49±0.07. The mean monthly crossing rate for all species at WCS3A was 0.56±0.04 during construction and 0.53±0.05 post-construction. A two-way ANOVA showed that there was no significant difference in crossing rate between WCS or between the during and post-construction periods on a monthly basis, for all species combined (Site: *F*_2_ *=* 0.338, *P =* 0.714; Period: *F*_1_ *=* 1.17, *P =* 0.282), and there was no significance found in the interaction of site and period (*F*_2_ *=* 0.170, *P =* 0.844) (S1 Fig). PERMANOVA results revealed that there were significant differences between the communities crossing between the three WCS (Pseudo-*F*_2_ *=* 12.79, *P≤*0.001) and between construction periods nested within the site (Pseudo-*F*_3_ *=* 3.29, *P≤*0.001; Fig 8). Further pairwise testing showed that the communities crossing at each individual WCS were significantly different between the two time periods (WCS1: *t =* 1.91, *P =* 0.010; WCS2: *t =* 1.63, *P =* 0.030; WCS3A: *t =* 1.73, *P =* 0.015). In a follow-up test for PERMDISP, it was found that dispersion among samples was significantly different between sites (*F*_2,75_ *=* 26.26, *P≤*0.001), but this was not true between the two construction periods (*F*_1,76_ *=* 2.19, *P =* 0.180; Fig 8). The results of the PERMANOVA were accepted, as a significant PERMDISP likely indicates a combination of position and dispersion in the data (Anderson et al. 2008).

Only raccoons used WCS1 to cross SH 100 during construction; raccoons, opossums, and bobcats all used WCS1 post-construction (Table 1). Similarly, at WCS2, only raccoons crossed during construction, and raccoons, opossums, and bobcats used WCS2 post-construction. Multiple species used WCS3A both during and after post-construction, and collared peccaries (*Pecari tajacu*) began to use WCS3A to cross post-construction (Table 1). Due to occurrences of ocelots being extremely rare (n = 1) within this period of this study, they were not included in any analyses.

A two-way ANOVA showed that the monthly species richness of wildlife crossing at WCS was significantly different between sites (*F*_2_ = 134.6, *P*≤0.001) and construction period (*F*_1_ = 18.2, *P*≤0.001; S2 Fig). There was no significant interaction between site and construction period (*F*_2_ = 0.42, *P* = 0.658). A post-hoc pairwise Tukey test showed that the mean monthly species richness of wildlife crossing was significantly higher at WCS3A than WCS1 (*P*≤0.001) and higher at WCS3A than WCS2 (*P*≤0.001), but no difference was found between WCS1 and WCS2 (*P* = 0.704; S2 Fig).

### WG and wildlife interactions

**Fig 9:**
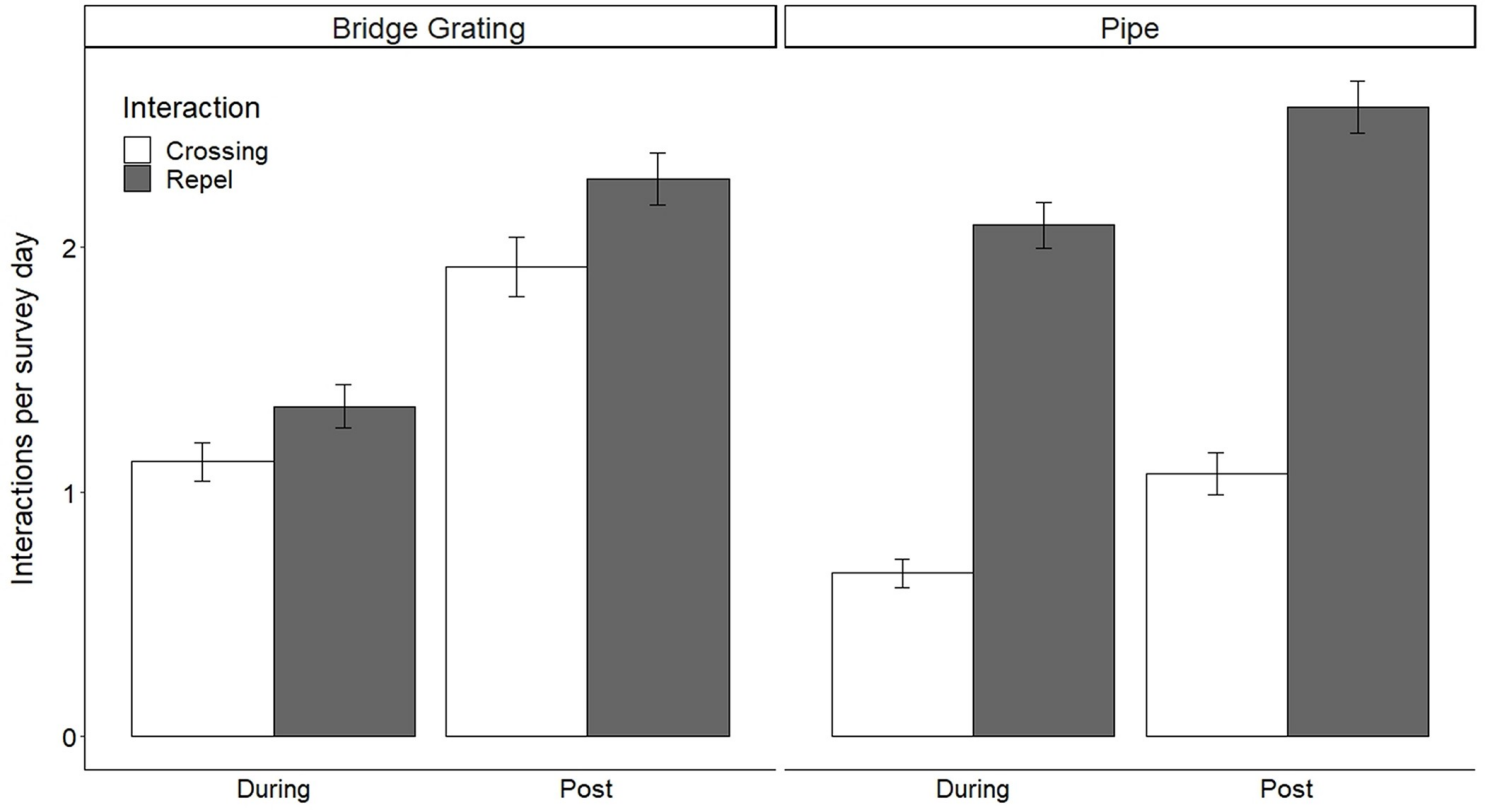
Bar graph showing mean number of repels and crossings per survey day ± standard error at pipe and bridge grating wildlife guards during construction (Apr 2017-May 2018) and post construction (May 2018-May 2019) along State Highway 100 in Cameron County, Texas. Repels and crossings per survey day were significantly different between pipe and bridge grating wildlife guards (*P* = 0.001) and significantly higher post construction (*P* = 0.002).

Cameras at WG were deployed for a total of 3,465 survey days during construction and 3,918 survey days post-construction at PWG and BGWG combined. For both PWG and BGWG combined, 2,570 interactions were recorded during construction, and 3,368 interactions were recorded post-construction (Table 3). At PWG during construction, a mean of 2.09±0.09 repels and a mean of 0.67±0.06 crossings were observed per survey day, and post-construction, 2.57±0.11 repels and 1.07±0.09 crossings occurred per survey day (Fig 9). At BGWG during construction, 1.35±0.09 repels and 1.22±0.08 crossings occurred per survey day, and post-construction, 2.28±0.11 repels and 1.92±0.12 crossings occurred per survey day. A PERMANOVA showed that the mean number of repels per survey day was significantly different between PWG and BGWG (*Pseudo-F*_1_ = 34.4, *P* = 0.001) and between during and after post-construction (*Pseudo-F*_1_ = 47.1, *P* = 0.002). There was a significant interaction between WG type and construction period (*Pseudo-F*_1_ = 10.2, *P* = 0.002). A PERMANOVA showed that mean crossings per survey day were significantly different between PWG and BGWG (*Pseudo-F*_1_ = 47.1, *P* = 0.001) and between during and post-construction (*Pseudo-F*_1_ = 27.9, *P* = 0.001). There was no significant interaction between WG type and construction period (*Pseudo-F*_1_ = 1.66, *P* = 0.195; Fig 9).

**Table 3:** Number of wildlife interactions determined by photo evaluation and count of different species detected by cameras at two wildlife guard (WG) types constructed by the Texas Department of Transportation to protect ocelots and other animals’ motility on State Highway 100 in Cameron County, Texas. Interactions observed at WG were compared during construction versus post construction. Monitoring was broken into two-time periods; during construction (April 2017—May 2018) and post construction (May 2018—May 2019).

WG	Interactions						Species Count					
	During			Post			During			Post		
	Repel	Crossing	Total	Repel	Crossing	Total	Repel	Crossing	Total	Repel	Crossing	Total
Bridge grating	1145	385	1530	1295	701	1996	17	11	17	18	14	18
Pipe	788	252	1040	939	433	1372	15	9	15	18	11	18

**Fig 10:**
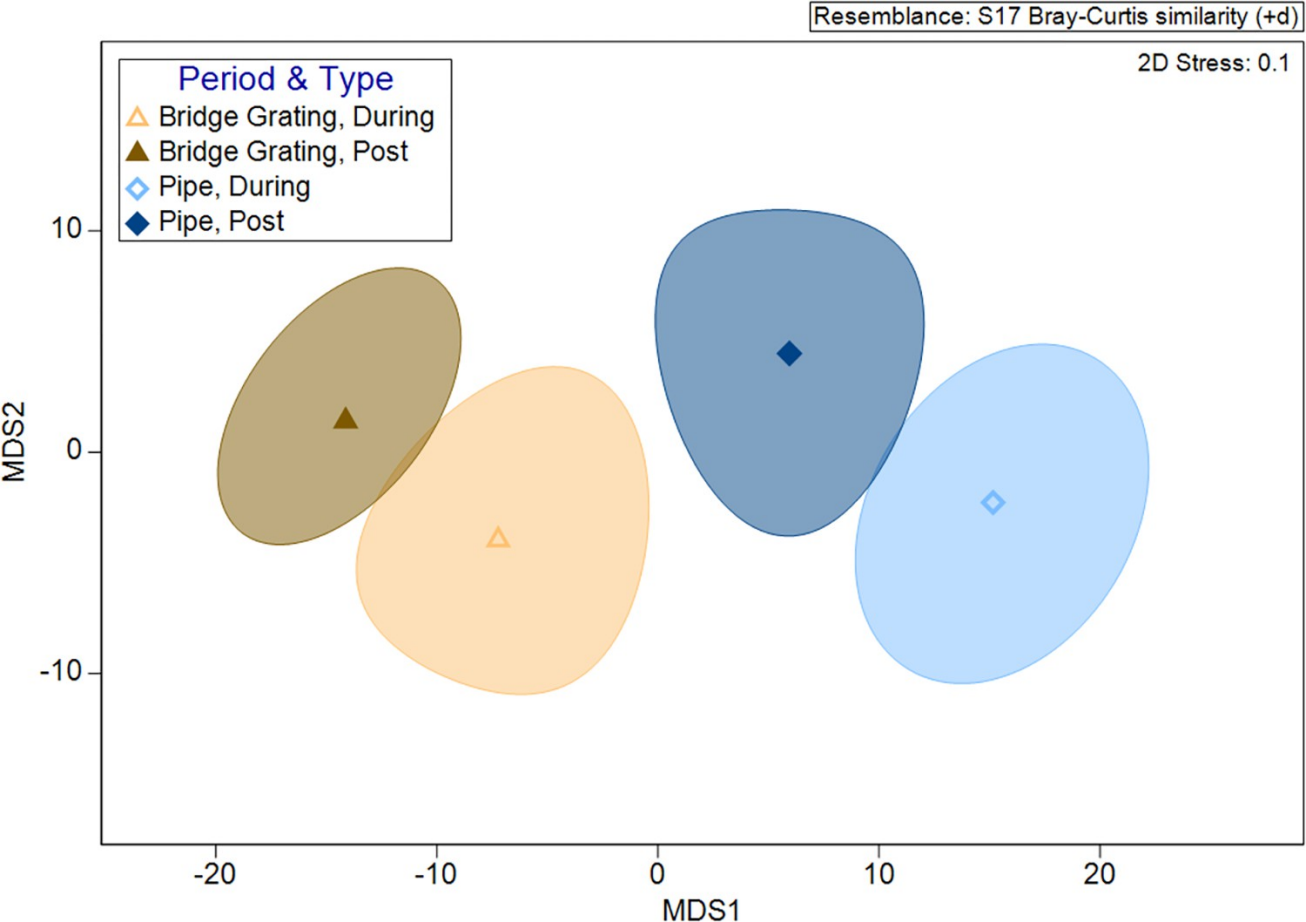
Bootstrapped metric multidimensional scaling (MDS) plot showing differences in monthly repel rates between communities observed at each wildlife guard type (*P* = 0.012) and between during construction (Apr 2017-May 2018) and post construction (May 2018-May 2019; *P* = 0.010) along State Highway 100 in Cameron County, Texas. Differences are indicated by physical distance between bootstrapped averages surrounded by 95% confidence areas.

A comparison of repel rates during construction and post-construction showed no significant difference (*W =* 7958, *P =* 0.195; S3 Fig), but there was a significant difference in repel rates between BGWG and PWG across both time periods, with greater repel rates at PWG (*W =* 6946, *P* = 004; S3 Fig). PERMANOVA results showed that the communities of species being repelled by WG were significantly different between WG types (Pseudo-*F*_1_ *=* 7.60, *P =* 0.012) and between the during and post-construction periods nested within WG type (Pseudo-*F*_2_ *=* 2.42, *P =* 0.010; Fig 10). Further pairwise testing showed no significant difference in communities between the during and post-construction periods at BGWG (*t =* 1.39, *P =* 0.085); however, there was a significant difference between communities during construction versus post-construction at PWG (*t =* 1.70, *P =* 0.022). Results of PERMDISP did not show any significant differences in the dispersion of samples between the two WG types (*F*_1,263_ *=* 0.68, *P =* 0.455) or between the during and post-construction periods (*F*_1,263_ *=* 1.22, *P =* 0.338; Fig 10).

SIMPER results showed that during construction and post-construction, the species that contributed the most to the community being repelled by PWG were raccoons, coyotes, black-tailed jackrabbits, opossums, eastern cottontails, white-tailed deer, striped skunks, domestic cats, and bobcats (Table 4). At BGWG, opossums, coyotes, raccoons, domestic cats and sheep, eastern cottontails, white-tailed deer, and striped skunks characterized the community of species being repelled during construction, which remained quite similar into the post-construction period (Table 4).

**Table 4:** Most abundant species being repelled at pipe wildlife guards (PWG) and bridge grating wildlife guards (BGWG), during and post construction, as a result of similarity percentage analysis of data collected from April 2017 to May 2019 on State Highway 100 in Cameron County, Texas. Average number of individuals denotes average number of individuals being repelled per month, and similarity percentage refers to the extent to which each species contributed to the similarities of communities observed crossing at each WCS and time period.

Type	During			Post		
	Species	Average no. individuals	Similarity percentage	Species	Average no. individuals	Similarity percentage
PWG	Raccoon	2.0	55.9	Opossum	4.0	40.3
	Coyote	1.1	20.3	Raccoon	0.6	16.0
	Black-tailed jackrabbit	0.6	11.0	Coyote	0.8	13.0
	Opossum	0.7	3.9	Black-tailed jackrabbit	0.9	10.6
	Eastern cottontail	0.4	2.8	Striped skunk	0.6	9.7
	White-tailed deer	0.3	1.8	Eastern cottontail	1.9	4.8
	Striped skunk	0.2	1.8	Domestic cat	0.3	2.0
	Domestic cat	0.3	1.3	White-tailed deer	0.3	1.5
	Bobcat	0.1	0.8	Bobcat	0.2	0.9
				Nine-banded armadillo	0.2	0.1
BGWG	Opossum	2.7	61.9	Opossum	5.3	68.6
	Coyote	0.6	14.3	Domestic cat	2.1	10.7
	Raccoon	0.4	8.6	Coyote	0.7	8.8
	Domestic cat	0.5	4.7	Striped skunk	0.6	5.5
	Domestic sheep	1.1	4.3	White-tailed deer	0.4	2.2
	Eastern cottontail	1.1	2.8	Raccoon	0.3	1.5
	White-tailed deer	0.2	1.4	Domestic sheep	0.6	1.1
	Striped skunk	0.3	1.2	Eastern cottontail	0.2	0.9

A two-way ANOVA showed no significant difference in monthly species richness between the PWG and BGWG (*F*_1_ = 1.72, *P* = 0.197); however, there was a significant difference between during and post-construction (*F*_1_ = 5.11, *P* = 0.029; S4 Fig). There was no interaction between WG type and construction period for monthly species richness (*F*_1_ = 0.21, *P* = 0.649).

### WCS structural and environmental factors

The global generalized linear model (GLM) had an R^2^ of 0.15 and showed significant negative relationships between counts of crossings and openness ratio (GLM*z*_4226_ = -17.36, *P≤*0.001), precipitation (GLM*z*_4226_ = -2.16, *P =* 0.031), length (GLM*z*_4226_ = -15.23, *P≤*0.001), and daily low temperature (GLM*z*_4226_ = -8.36, *P≤*0.001; Table 5). There were significant positive relationships between counts of crossings and WCS height (GLM*z*_4226_ = 9.67, *P≤*0.001) and distance to vegetation (GLM*z*_4226_ = 9.60, *P*≤0.001; Table 5). Daily high temperatures were found to be correlated with daily lows (*r* = 0.88), as was width with openness ratio (*r* = 0.99), so daily high temperatures and width were omitted from the model.

**Table 5:** Results of global generalized linear model testing counts of all species crossings against structural characteristics and landscape variables in the post construction period (May 2018-May 2019) in Cameron County, Texas.

Factor	Estimate ± SE	*Z*	P
*Intercept	1.33 ± 0.13	10.15	≤0.001
*Openness	-5.29 ± 0.30	-17.36	≤0.001
*WCS height	1.19 ± 0.12	9.67	≤0.001
*WCS length	-0.05 ± 0.003	-15.23	≤0.001
*Precipitation	-0.06 ± 0.03	-2.16	0.031
*Daily low temperature	-0.02 ± 0.002	-8.36	≤0.001
*Distance to vegetation	0.01 ± 0.001	9.60	≤0.001

*Statistical significance within 95% confidence intervals.

The best-fitting model for bobcats included daily low temperature and precipitation as factors, and there was a significant negative relationship between counts of bobcat crossings and daily low temperature (GLM*z*_266_ = -2.03, *P =* 0.042; Table 6). This model had a McFadden’s pseudo-R^2^ of 0.02.

The best-fitting model for coyote crossings included only WCS height, and there was a significant negative relationship between counts of coyote crossings and this factor (GLM*z*_196_ = -7.26, *P≤*0.001; Table 6). This model had a McFadden’s pseudo-R^2^ of 0.36.

**Table 6:** Results of best-fitting generalized linear models resulting from the dredge function in R, testing binomial counts of crossings of the five most frequently observed species against environmental factors, from data collected post construction (May 2018—May 2019) in Cameron County, Texas.

Species	Factor	Estimate ± SE	*Z*-value	*P*
Bobcat	*Intercept	2.26 ± 0.70	3.22	≤0.001
	Precipitation	-0.27 ± 0.15	-1.73	0.083
	*Daily low temperature	-0.04 ± 0.02	-2.03	0.042
Coyote	*Intercept	7.99 ± 1.0	7.93	≤0.001
	*WCS height	-5.04 ± 0.69	-7.26	≤0.001
Raccoon	*Intercept	1.86 ± 0.23	8.01	≤0.001
	*Openness ratio	-3.28 ± 0.88	-3.73	≤0.001
	*Distance to vegetation	-0.01 ± 0.004	-2.54	0.011
Opossum	*Intercept	7.30 ± 0.63	11.54	≤0.001
	*Openness ratio	-4.78 ± 1.55	-3.08	0.002
	WCS height	-0.99 ± 0.70	-1.42	0.154
	*WCS length	-0.07 ± 0.02	-3.12	0.002
	*Precipitation	-0.27 ± 0.12	-2.31	0.021
	*Daily low temperature	-0.04 ± 0.01	-3.78	≤0.001
	*Vegetation distance	0.05 ± 0.01	5.42	≤0.001
Nine-banded armadillo	*Intercept	4.27 ± 0.67	6.38	≤0.001
	*WCS height	-3.49 ± 0.51	-6.89	≤0.001

*Statistical significance within 95% confidence intervals.

The best-fitting raccoon model included openness ratio and distance to vegetation as factors. There were significant negative relationships between counts of raccoon crossings and openness ratio (GLM*z*_433_ = -3.73, *P≤*0.001) and vegetation distance (GLM*z*_433_ = -2.54, *P =* 0.011; Table 6). This model had a McFadden’s pseudo-R^2^ of 0.04.

The best-fitting model for opossum crossings included openness ratio, WCS height, WCS length, precipitation, daily low temperature, and distance to vegetation as factors. There were significant negative relationships between counts of opossum crossings and openness ratio (GLM*z*_1258_ = -3.08, *P* = 0.002), WCS length (GLM*z*_1258_ = -3.12, *P* = 0.002), precipitation (GLM*z*_1258_ = -2.31, *P =* 0.021), and daily low temperature (GLM*z*_1258_ = -3.78, *P≤*0.001; Table 6). There was a significant positive relationship between opossum crossings and vegetation distance (GLM*z*_1258_ = 5.42, *P≤*0.001). This model had a McFadden’s pseudo-R^2^ of 0.19.

Lastly, the best-fitting nine-banded armadillo model, as a result of model selection, only included WCS height as a factor, and there was a significant negative relationship between counts of armadillo crossings and WCS height (GLM*z*_325_ = -6.89, *P≤*0.001; Table 6). This model had a McFadden’s pseudo-R^2^ of 0.19.

### Water levels at WCS3

**Fig 11:**
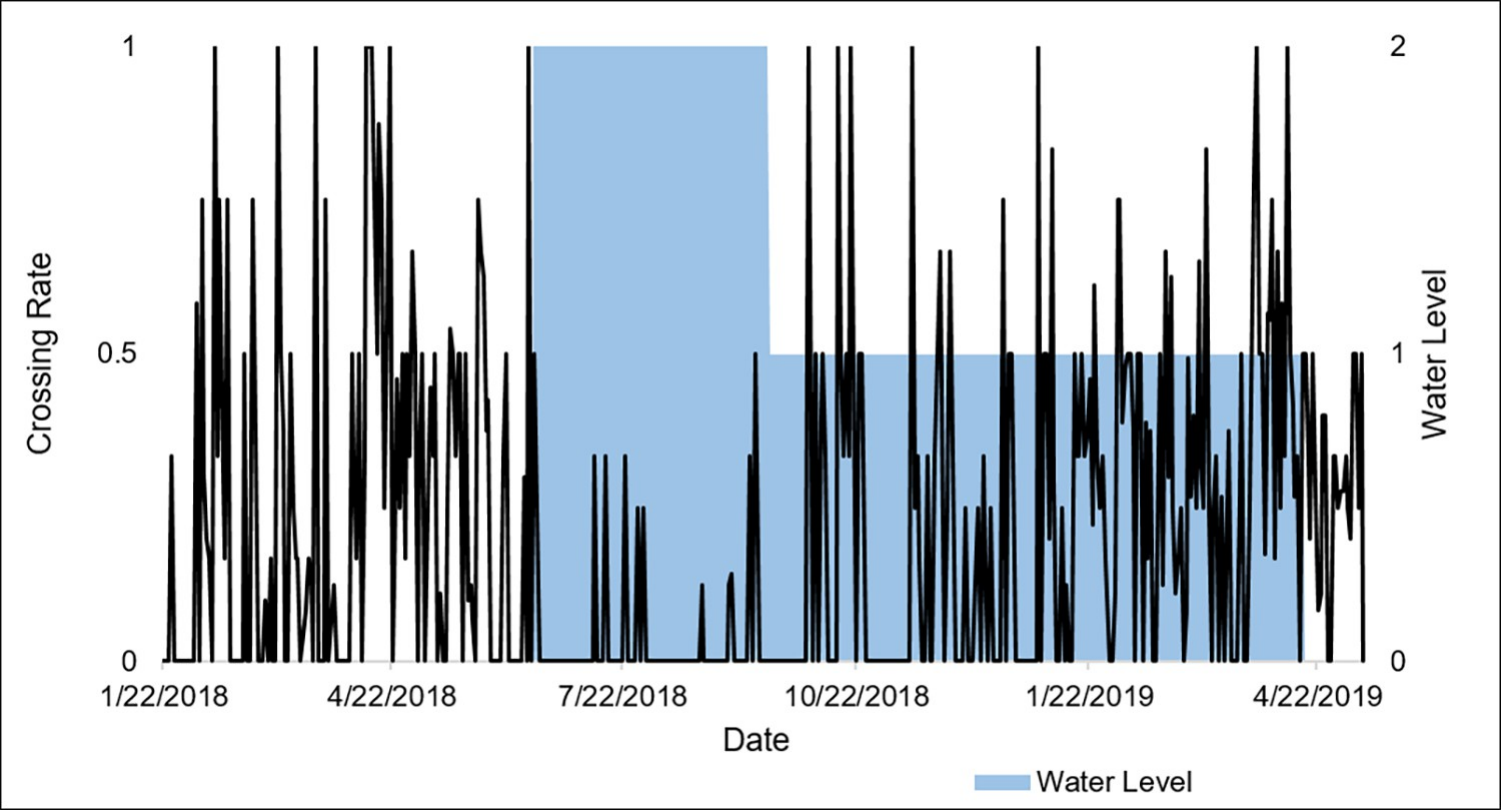
Daily crossing rates (Σcrossings/Σtotal occurrences) of all wildlife compared to three assigned water levels at WCS3 for data collected from January 2018 to May 2019 in Cameron County, Texas. Water levels indicate little to no water (0); intermediate pooling of water (1); full of water (2).

A Kruskal-Wallis’s test determined that there was a significant difference in daily crossing rates at three different water levels at WCS3 (χ^2^_2_ = 57.19, *P≤*0.001; Fig 11). A Dunn test using a Bonferroni correction showed that there was no difference in daily crossing rates between water levels 0 and 1 (*Z =* 0.25, *P =* 1.00), but there were significant differences in daily crossing rates between water levels 1 and 2 (*Z =* 6.93, *P≤*0.001) and levels 0 and 2 (*Z =* 6.89, *P≤*0.001; Fig 11).

## Discussion

### Wildlife interaction with road mitigation structures

The results of this study did not support the hypothesis that crossing rates for all species would increase after construction had ended. While not statistically significant (*P* = 0.282), the average crossing rate decreased between the during and post-construction periods for WCS1, WCS2, and WCS3A, which may be due to the overall reduction in activity at WCS1 and WCS2, as shown in Fig 6 as well as the construction of two new structures, WCS3 and WCS4. This does not, however, take the communities using the WCS into consideration. The PERMANOVA results showed that not only were the communities crossing at each WCS different, but they also differed between the two construction periods at each location.

The hypothesis that species richness of wildlife crossing at WCS post-construction would increase was supported by findings from analyses. Similarity percentages (SIMPER) provided further detail into changes in species activity and showed that each species that used each WCS showed its own increase, decrease, or lack of change in use from during to post-construction. For example, raccoon crossings decreased, while bobcat and opossum crossings increased at both WCS1 and WCS2. The overall use of WCS3A increased and increases in the use of bobcats, collared peccaries, and Texas indigo snakes at WCS3A contributed to the change in the community (Table 1). It is likely that the increase in the use of WCS3A was, in part, due to the completion of exclusionary fencing along with Texas SH 100. This would be consistent with findings that adding fencing along the right-of-way helps increase wildlife crossings via underpasses and overpasses [7–11].

Increases in WCS use may also be due to habituation [48]. While WCS3A had been in place the longest out of the three WCS analyzed on SH 100 and already experienced relatively high wildlife traffic, species such as collared peccary were likely still becoming habituated to this structure. Collared peccaries had not been observed crossing at WCS3A at any point during the monitoring period prior to the completion of construction. There had been many observations of entry-and-exit and approach behaviors, but there were no full crossings by collared peccaries observed until four months post-construction. Use by javelina four months after construction demonstrates that even if structures are not used immediately, users may continue to change over time. This was also seen in elk using newly constructed WCS in central Arizona [12] and in squirrel gliders, using canopy bridges in southeast Australia [49]. van der Grift and van der Ree [19] hypothesized that WCS use would increase in the future until the species using the WCS closely resembles the community surrounding the structure.

Habituation to WCS1 and WCS2 by bobcats and opossums likely occurred, as the newly widened catwalks provided a dry path to allow them to cross. Other studies have also shown that similar dry ledges have been beneficial in facilitating wildlife crossings [50, 51]. Our observations, as well as those of the aforementioned studies, provide evidence that dry pathways are an important consideration in the construction and configuration of WCS that may have a seasonal or permanent flow of water. It is unclear what is the likely cause of the decrease in raccoon activity between the two time periods at both WCS1 and WCS2. As these WCS have a nearly constant flow of water, the presence of aquatic animals, such as small fish, crabs, and crayfish have provided foraging opportunities for raccoons. Foraging was a frequently observed behavior of raccoons in and around these structures, mostly prior to completion of construction; however, raccoon activity declined after flooding at WCS1 and WCS2 resulting from heavy rainfall that occurred post-construction. Furthermore, increased bobcat activity at WCS1 and WCS2 may have influenced the decrease in raccoon activity, as raccoons may have been avoiding predators.

The hypothesis that repel rates at WG would increase from during construction to post-construction was not supported. Even with the lack of statistical significance, though, there was a slight decrease in repel rates and an increase in crossing rates. These results are similar to what was observed for carnivore species encountering and crossing WG in northwestern Montana [16]. The Wilcoxon rank-sum test did not take repel rates of the communities interacting with these structures into account. PERMANOVA did show a difference in repel rates of communities between them during and post-construction periods for both PWG and BGWG combined, and the post-hoc pairwise test did reveal a difference between the construction periods for PWG but not for BGWG.

The hypothesis that species richness would increase post-construction at WG was supported. Further insight into changes in species being repelled was provided by SIMPER analysis. The SIMPER analysis showed that at PWG, repels of raccoons decreased, and repels of opossums increased from during to post-construction, suggesting that these two species were the main drivers of differences observed at PWG (Table 4). At BGWG, increases in repel behavior by opossum and domestic cats likely drove the differences in communities from during to post-construction. It is probable that wildlife started to become habituated to WG and learned how to cross. Not only were more wildlife approaching WG to inspect and test them out, but they eventually became familiar enough with the structures to cross over or through them. VanCauteren et al. [52] also documented habituation to deer guards by white-tailed deer and reported deer jumping across and using crossbars to walk across the guards. In the present, the same behaviors were observed of the two large ungulate species along with Texas SH 100, white-tailed deer and nilgai (*Boselaphus tragocamelus*), though crossing events were infrequent compared to repel events. On several occasions, attempts to jump across WG were unsuccessful, which was likely due to the length of the WG. Sebesta et al. [14] also reported deer guard length as an important factor in preventing deer from crossing these structures.

An important finding of this study is that bobcat, coyote, and opossum crossings at WG noticeably increased and are consistent with the findings of Allen et al. [16] that WGs are not substantial barriers to multiple carnivore species, including bobcats and coyotes. On only one observed occasion, an individual ocelot encountered a PWG and crossed into the roadway without any apparent hesitation. This suggests that WG may not be an effective road mitigation strategy for ocelots or other felids. Habituation was also demonstrated by more than just crossing behaviors. At PWG, several species, including domestic cats (*Felis catus*), opossums, long-tailed weasels (*Mustela frenata*), and several squamate species, were observed slipping between pipes into the excavated areas increasingly in the post-construction period. This was likely due to frequent small rodent activity in and around WG as a source of prey, though it is speculated, based on observations of individuals spending extended periods of time below the WG, that some wildlife sought these excavated areas as cover.

Wildlife guards installed along with Texas SH 100 were designed to deter wildlife from entering the road and to allow the passage of emergency vehicles. The PWGs were constructed with 7.6 cm pipe and were supported by 15.2 cm wide steel beams. The large size of the pipes and the underlying beams appeared to provide ample footing for animals to walk across PWG (Fig 3). Despite expectations that PWG would be effective in preventing crossings, several bobcats and one ocelot crossed into the roadway using the crossbars as footpaths. Another unintentional footpath that was created was the cement pad between the side panel fencing and the WG. Although a similar design to BGWG was reported as the most effective design for deterring Florida Key deer (*Odocoileus virginianus clavium*) [15], this does not seem to be the case for coyotes, opossums, domestic cats, or domestic sheep along SH 100.

The original hypotheses that crossing rates at WCS would increase post-construction (1) and that repel rates at both WG types would increase post-construction (3), were not supported by the findings of this study. Habituation of individuals over time likely played a factor in driving the observed WG results. However, the data also showed increases in species richness over time using the WCS and being repelled at WG.

### Influence of structural characteristics and environmental factors on wildlife

All factors in the global GLM were determined to be predictors of overall wildlife crossings, but these varied by species. There was a negative relationship between counts of wildlife crossings and increases in the WCS openness ratio. This negative association with the openness ratio was also found to be true of black bears and mountain lions in Banff National Park, Canada [21] and small mammals and mustelids in Northwest Spain [53]. When going further to examine the effects of individual dimension parameters of WCS on wildlife crossings, it was found that crossings increased with increases in WCS height but decreased with increases in WCS length. Mule deer use of underpasses in Utah showed the same relationship with height and length [20], though Clevenger and Waltho [21, 23] found no significant relationship between carnivore underpass usage and these individual dimensions.

Successful crossings of wildlife decreased with increases in precipitation. When examining the effects of other weather variables, such as temperature, it was found that increases in minimum temperatures, typically nighttime temperatures, had a negative effect on crossings as well. Surprisingly, wildlife crossings did not increase as the distance from the nearest large vegetation patch to the WCS entrance decreased, as was described of mustelids in the Netherlands [27]. Although statistically significant factors, global GLM estimates of daily low temperature, vegetation distance, and WCS length were low, thus suggesting low chances of influencing wildlife crossings. The likelihood of precipitation influencing wildlife crossings was also minimal. The structural factors for WCS openness ratio and WCS height were significant for several species models and are important features to consider in future mitigation projects. While the global model provides good baseline information, it is important to examine the influences of structural characteristics and environmental factors on individual species’ use of WCS. Not all factors in the global model were important predictor variables in each species’ use of WCS.

For bobcats, the best predictors of their crossing were the weather parameters. Only daily low temperature was significant, but the estimate for this factor suggested that the chances of crossings changing due to temperature were relatively low. Bobcats are described as habitat generalists and are highly adaptable, so it is possible that cover, in the form of either WCS or vegetation, may not have been an important factor influencing their use of WCS [54]. Murphy-Mariscal et al. [55] also found that structural characteristics played a minor role in bobcat use of underpasses, and bobcat use was more related to human activity and prey availability, which were not measured in this study. WCS height was shown to be the only significant variable influencing coyote crossings, and the estimate suggested a high likelihood of a decrease in crossings as height increased. It is unclear why WCS height would influence coyote use of WCS, as there is scarce literature that suggests this. Coyotes have been documented using a wide range of WCS [55, 56], and multiple road mitigation handbooks suggest that most types of underpasses are suitable for coyotes [4, 57]. It is possible that coyotes followed smaller animals, or potential prey, into WCS with shorter heights, but such interactions were not well documented in this study.

Height was also shown to affect armadillo use of WCS. The use of WCS with lower heights by armadillos may be explained by their tendency toward brushy areas [54, 58], and WCS with lower heights, such as WCS3A, may be a potential substitute for cover. Additionally, armadillos were only observed at WCS3A and WCS3, which are the only WCS within the study area with natural soil substrate. Armadillos tend to roll in wet mud to cool off during warm temperatures, and when soils harden, this provides opportunities for armadillos to forage for insects [54]. Both of these behaviors were observed in this dataset and may be influencing factors in armadillo use of WCS.

Increases in both openness ratio and distance to vegetation had significant negative effects on raccoon crossings, but the low estimate of the distance to vegetation suggested that it had a relatively low likelihood of causing raccoon crossings to decrease. Consistent with the findings of Mata et al. [53] that smaller animals tend to choose smaller culverts the openness ratio had a greater likelihood of causing raccoon crossings to decrease. Raccoons crossed at all five WCS. Despite their use of WCS1 and WCS2 having decreased from during construction to post-construction, raccoons crossed the most at these two WCS post-construction, followed by WCS3A. Their high usage of WCS1 and WCS2 may have been due to the regular flow of water through these structures and raccoons’ association with water [54]. Raccoons’ use of WCS3A may have been due to the age of the structure and their established familiarity with this WCS.

The opossum model included all the global model variables. This may be due to opossum crossings comprising nearly a third of total post-construction wildlife crossings across all WCS. Similar trends were observed in terms of directionality and significance with these variables, with the exception of WCS height, which in the opossum model, had a statistically insignificant negative effect on opossum crossings. The estimate for the openness ratio from the model suggests that this factor has a high likelihood of causing a decrease in opossum crossings as the openness ratio increases. This finding is also consistent with that of Mata et al. [53], as opossums’ smaller body size may be related to their crossing through smaller WCS. The estimate for precipitation suggests a moderate likelihood that this factor negatively influences opossum crossings. While high amounts of precipitation resulting in WCS flooding may have temporarily kept opossums from using WCS, it is also possible that pooled water following rain resulted in a lesser need for opossums to go in search of a water source. Gagnon et al. [12], Clevenger and Waltho [23], Grilo et al. [28], Mata et al. [53], and Ng et al. [56] reported WCS dimensions as some of the most important factors influencing certain species’ crossings; however, this was not always the case in this study. As previously noted, bobcats in this study were not influenced in their crossing frequency by structural characteristics. Contrary to the above-cited studies, Murphy-Mariscal et al. [55] found that structural dimensions of underpasses were minorly influential in carnivore use, but instead, other factors such as habitat fragmentation and human activity appeared to have had an impact on bobcat and coyote use.

Many studies that attempt to describe factors that affect wildlife use of WCS include variables other than structural dimensions, such as noise [23, 59], surrounding habitat type and quality [28, 56], proximity to other WCS [21], human activity [12, 23, 55], proximity to towns [21, 34], the flow of water through underpasses [26, 60], and existing wildlife home ranges and life history [53, 60, 61]. The results of this study were likely influenced by several of these factors, and it may be beneficial to include them in future aspects of this project. Furthermore, the low pseudo-R^2^ values for most of the models in this study suggest that may be the case.

Another variable that has been reported to influence wildlife use of underpasses is flooding [26, 60]. For this study, the hypothesis that wildlife crossing rates would decrease during periods of flooding at WCS3 was partially supported. When flooding was at its highest, crossing rates for all species dropped significantly. As water levels receded, crossing rates increased. A pathway developed along the edges of the water, which was concentrated toward the center and low point of WCS3, and this allowed smaller species, such as opossums, raccoons, and cottontails to pass under the road. White-tailed deer eventually became accustomed to the shallow, pooled water and were able to cross through the WCS as well. This is consistent with the findings of Craveiro et al. [26] and Serronha et al. [60] that intermediate levels of water in underpasses pose a minimal barrier to wildlife. Given this, it is important to consider WCS3’s construction flaw in that water does not drain away on its own. In order to reach an intermediate level of water following significant flooding, this underpass must be partially drained by pumping out the water. This restores function to WCS3, but if personnel are not immediately available to complete this task, then wildlife movement will be hindered.

WCS1, WCS2, and WCS3A have also experienced flooding. In some cases, the water level has been high enough to completely bar animals from approaching or entering. At more intermediate water levels, wildlife has been observed continuing to use WCS, including at WCS3A where a dry pathway is not available. On several occasions, raccoons, coyotes, and even bobcats have been observed using WCS3A at chest-deep levels of flooding. At WCS1 and WCS2, the catwalks provide a dry path for wildlife to use. The major difference between these WCS and WCS3, however, is that water drains away quickly, and flooding is short-lived. Because of this, there was not enough data to examine the effects of flooding on wildlife crossing rates at these WCS in this study, though, with several more years of data, it may be a possibility. Several more years of monitoring WCS would elucidate the more telling relationships between environmental factors and wildlife crossings. Gagnon et al. [12] stress the importance of long-term studies to take wildlife habituation into consideration and to better understand temporal changes in WCS use. Patterns of seasonality, of both wildlife activity and weather, could be revealed by continuing this study beyond one year.

## Conclusion

The results of this study show that differential wildlife use of WCS was affected by one or more factors (e.g., structural dimensions, distance to nearby vegetation, water presence, etc.), and as such, it is recommended that these factors be considered in species-specific approaches prior to the construction of mitigations structures. Although road mitigation structures on SH 100 were constructed with the intent to reduce road mortality and improve connectivity for ocelots, it is clear that a wide diversity of other species benefitted from these structures. Moreover, this short snapshot of the lives of these structures may not be indicative of potential future use of WCS by ocelots. Future monitoring of these structures could address wildlife habituation to WCS and other structures and would also be beneficial for documenting patterns of habituation, seasonality, other environmental factors. This work will add to a global effort to understand the efficacy of a wide variety of road mitigation measures and successfully manage the negative impacts that roads have on wildlife. Furthermore, this research and its implications may be applied in similar ecological systems in order to conserve wildlife and mitigate roads.

## Supporting information

S1 Fig Bar graph showing mean monthly crossing rates ± standard error of all species combined at each wildlife crossing structure (WCS) during construction (January 2017-May 2018) and post construction (May 2018-May 2019) along State Highway 100 in Cameron County, Texas. Crossing rates were not significantly different between WCS1, WCS2, and WCS3A (*P* = 0.714) orbetween during construction and post construction (*P* = 0.282).
(PDF)

S2 Fig Bar graph showing mean monthly species richness ± standard error for each wildlife crossing structure (WCS) during construction (Jan 2017-May 2018) and post construction (May 2018-May 2019) along State Highway 100 in Cameron County, Texas. Species richness was significantly higher at WCS3A (*P*≤0.001) and significantly higher post construction (*P*≤0.001).
(PDF)

S3 Fig Bar graph showing mean monthly repel rates ± standard error of all species combined at pipe and bridge grating wildlife guards during construction (Apr 2017-May 2018) and post construction (May 2018-May 2019) along State Highway 100 in Cameron County, Texas. Repel rates were significantly higher at pipe wildlife guards (*P* = 0.004) but were not significantly different between during construction and post construction (*P* = 0.195).
(PDF)

S4 Fig Bar graph showing mean monthly species richness ± standard error at pipe and bridge grating wildlife guards during construction (Apr 2017—May 2018) and post construction (May 2018—May 2019) along State Highway 100 in Cameron County, Texas. Species richness was not significantly different between pipe and bridge grating wildlife guards (*P* = 0.197) but were significantly higher post construction (*P* = 0.029).
(PDF)

S1 Table Type and dimensions (width, height, and length in meters) of wildlife crossing structures (WCS) constructed by the Texas Department of Transportation to protect ocelots and other animals’ mortality along State Highway 100 in Cameron County, Texas. All structures were monitored using remote cameras, and data were collected at WCS from January 2017 to May 2019. Openness ratio was calculated as width × height / length.
(DOCX)

S2 Table List of species and species group recorded interacting with wildlife crossing structures and wildlife guards constructed by the Texas Department of Transportation. Structures were constructed to protect ocelots and other animals’ mortality on State Highway 100 in Cameron County, Texas, between January 2017 and May 2019.
(DOCX)

S3 Table Descriptive statistics, including mean, standard deviation, and range, of quantitative factors being tested in the generalized linear model for data collected from May 2018 to May 2019 on State Highway 100 in Cameron County, Texas. (DOCX)

S4 Table Most abundant species crossing at wildlife crossing structure (WCS) 3 and WCS4 post construction, based on similarity percentage analysis of data collected from May 2018 to May 2019 in Cameron County, Texas. Average number of individuals denotes average number of individuals crossing per month, and percent (%) contributed refers to the extent to which each species contributed to the clustering of the species observed crossing within each group.
(DOCX)
