# Supplementary material for: Road mitigation structures designed for Texas ocelots: Influence of structural characteristics and environmental factors on non-target wildlife usage
Source: PLoS One. 2024 Jul 22;19(7):e0304857. doi: 10.1371/journal.pone.0304857 (PMC11262682; doi:10.1371/journal.pone.0304857)
Supplement: S1 Fig — Crossing rates were not significantly different between WCS1, WCS2, and WCS3A (P = 0.714) orbetween during construction and post construction (P = 0.282). (PDF) [file pone.0304857.s001.pdf]

Supplementary Figure 1

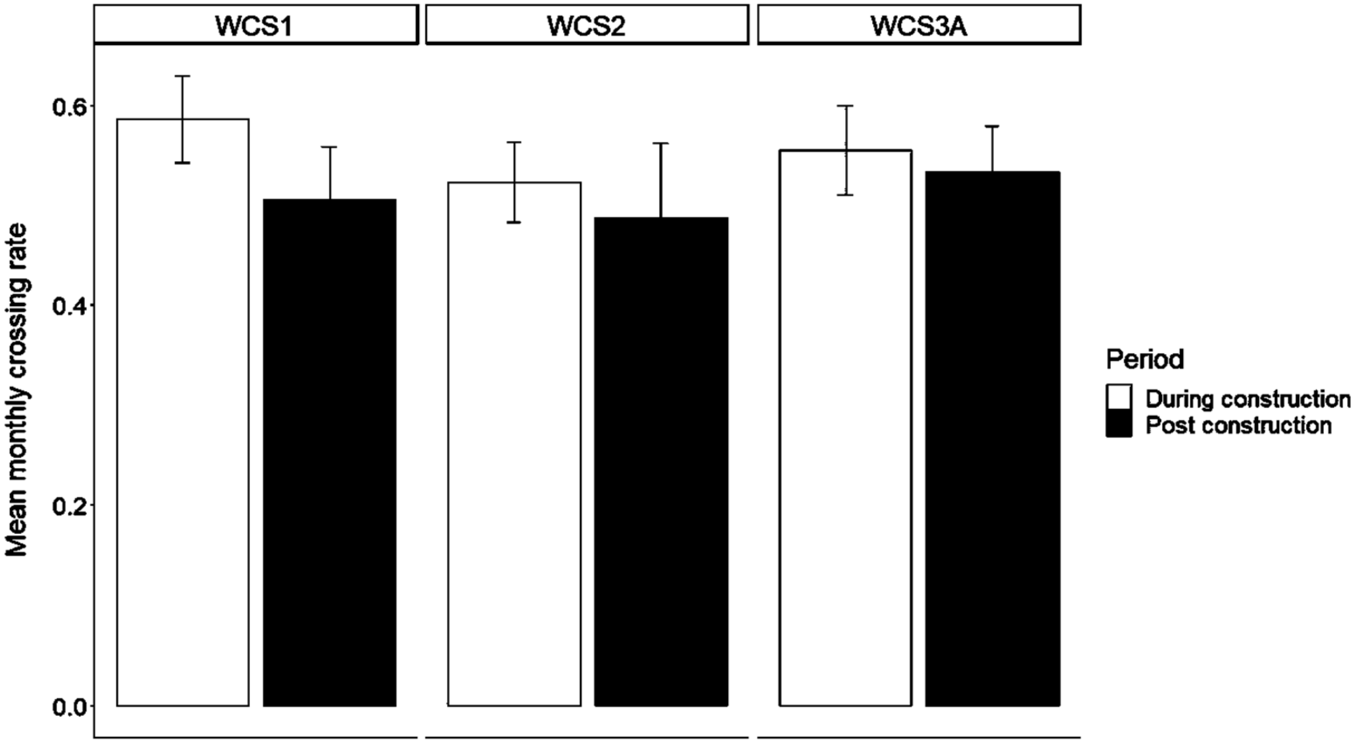

Supplementary 1. Bar graph showing mean monthly crossing rates ± standard error of all species combined at each wildlife crossing structure (WCS) during construction (January 2017-May 2018) and post construction (May 2018-May 2019) along State Highway 100 in Cameron County, Texas. Crossing rates were not significantly different between WCS1, WCS2, and WCS3A (P=0.714) or between during construction and post construction (P=0.282).
