# Supplementary material for: Road mitigation structures designed for Texas ocelots: Influence of structural characteristics and environmental factors on non-target wildlife usage
Source: PLoS One. 2024 Jul 22;19(7):e0304857. doi: 10.1371/journal.pone.0304857 (PMC11262682; doi:10.1371/journal.pone.0304857)
Supplement: S2 Fig — Species richness was significantly higher at WCS3A (P≤0.001) and significantly higher post construction (P≤0.001). (PDF) [file pone.0304857.s002.pdf]

Supplementary Figure 2

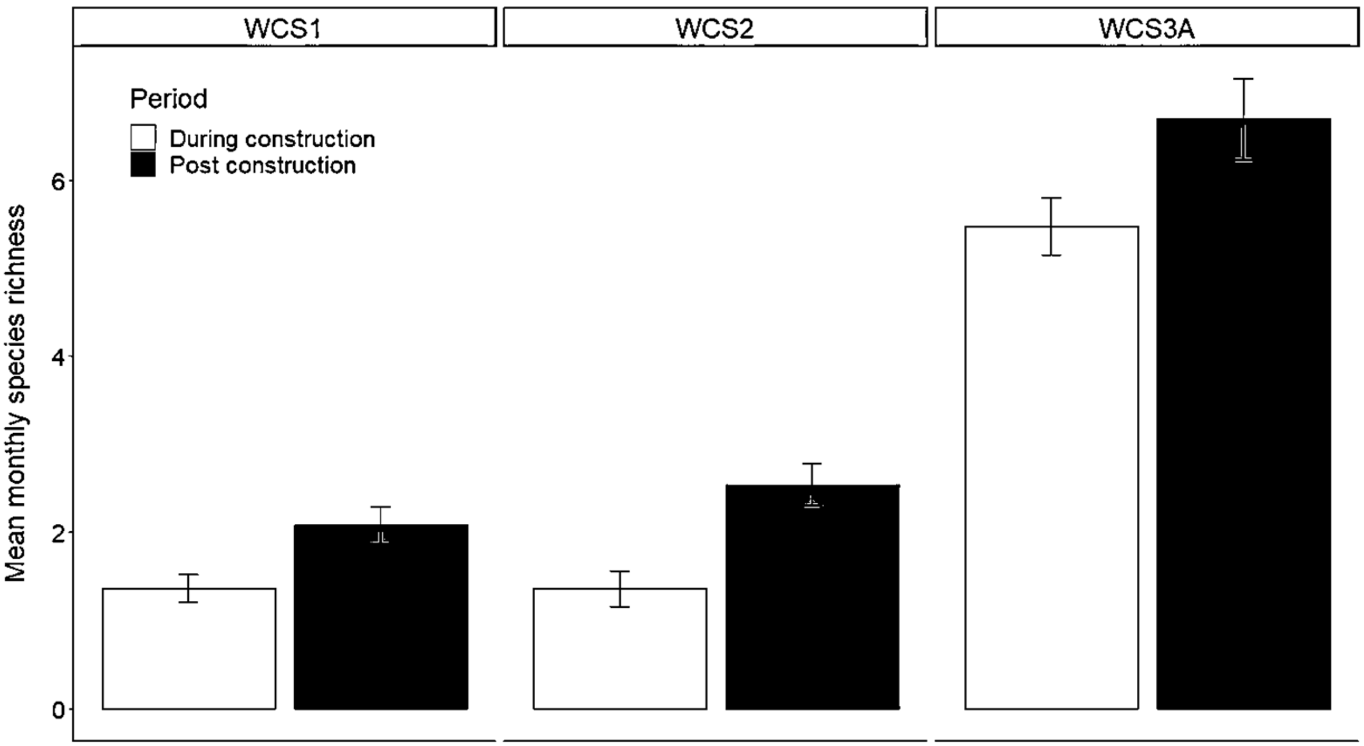

Supplementary Figure 2. Bar graph showing mean monthly species richness ± standard error for each wildlife crossing structure (WCS) during construction (Jan 2017-May 2018) and post construction (May 2018-May 2019) along State Highway 100 in Cameron County, Texas, USA. Species richness was significantly higher at WCS3A ($P \leq 0.001$) and significantly higher post construction ($P \leq 0.001$).
