# Supplementary material for: Road mitigation structures designed for Texas ocelots: Influence of structural characteristics and environmental factors on non-target wildlife usage
Source: PLoS One. 2024 Jul 22;19(7):e0304857. doi: 10.1371/journal.pone.0304857 (PMC11262682; doi:10.1371/journal.pone.0304857)
Supplement: S3 Fig — Repel rates were significantly higher at pipe wildlife guards (P = 0.004) but were not significantly different between during construction and post construction (P = 0.195). (PDF) [file pone.0304857.s003.pdf]

Supplementary Figure 3

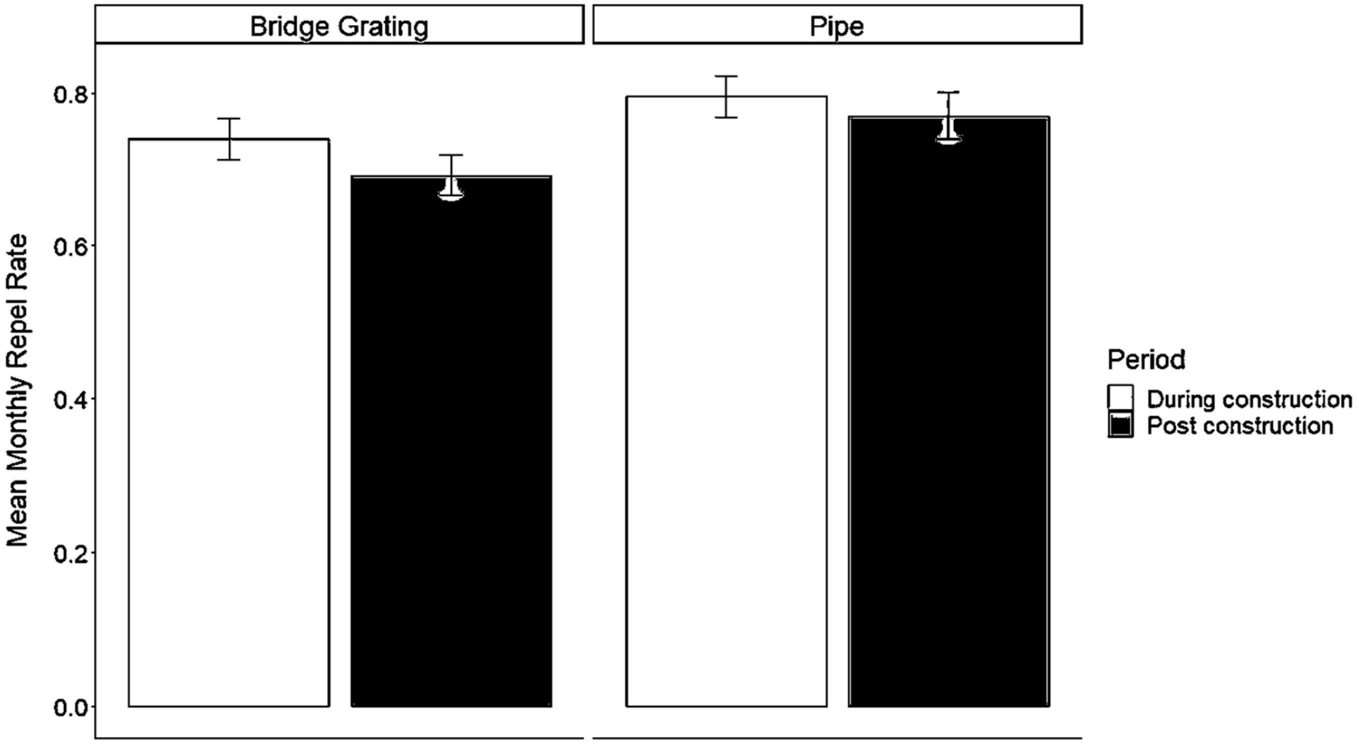

Supplementary Figure 3. Bar graph showing mean monthly repel rates ± standard error of all species combined at pipe and bridge grating wildlife guards during construction (Apr 2017-May 2018) and post construction (May 2018-May 2019) along State Highway 100 in Cameron County, Texas. Repel rates were significantly higher at pipe wildlife guards (*P*=0.004) but were not significantly different between during construction and post construction (*P*=0.195).
