# Supplementary material for: Road mitigation structures designed for Texas ocelots: Influence of structural characteristics and environmental factors on non-target wildlife usage
Source: PLoS One. 2024 Jul 22;19(7):e0304857. doi: 10.1371/journal.pone.0304857 (PMC11262682; doi:10.1371/journal.pone.0304857)
Supplement: S4 Fig — Species richness was not significantly different between pipe and bridge grating wildlife guards (P = 0.197) but were significantly higher post construction (P = 0.029). (PDF) [file pone.0304857.s004.pdf]

Supplementary Figure 4

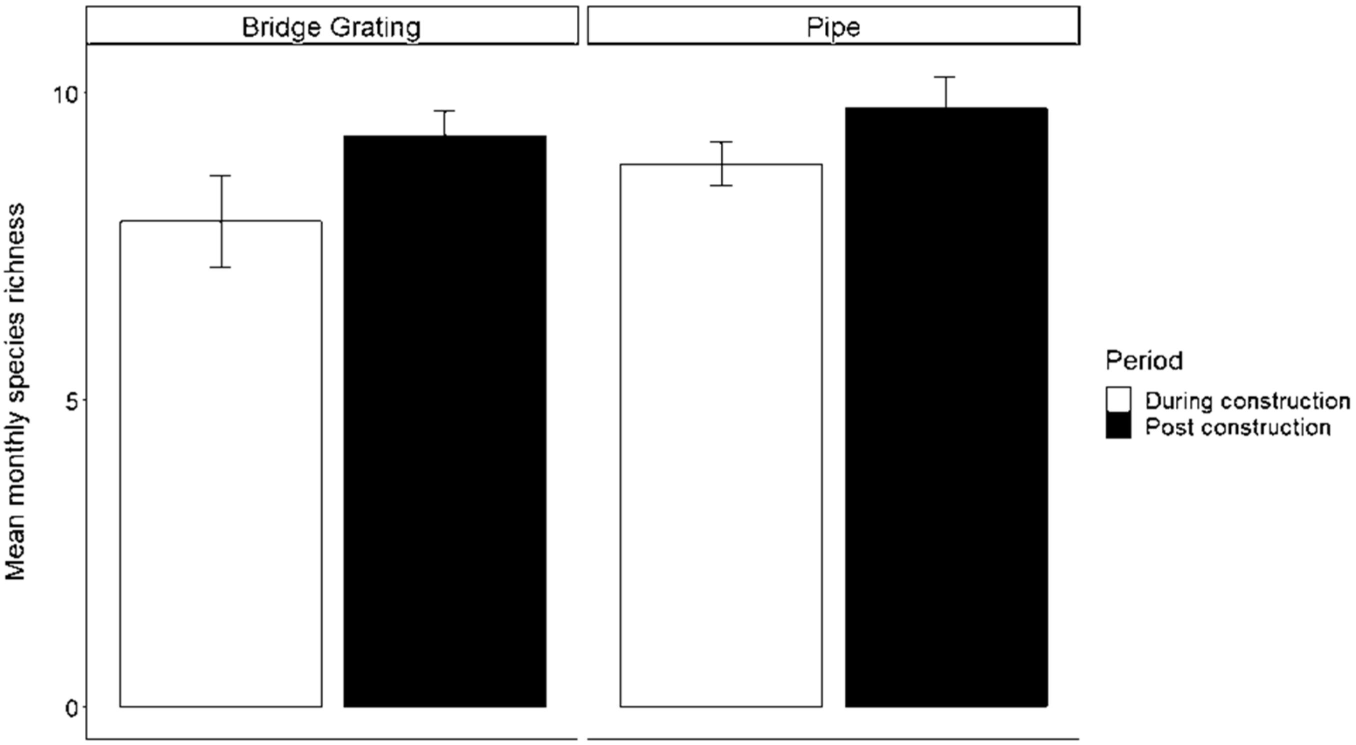

Supplementary Figure 4. Bar graph showing mean monthly species richness ± standard error at pipe and bridge grating wildlife guards during construction (Apr 2017 - May 2018) and post construction (May 2018 - May 2019) along State Highway 100 in Cameron County, Texas, USA. Species richness was not significantly different between pipe and bridge grating wildlife guards (*P*=0.197) but were significantly higher post construction (*P*=0.029).
