# Supplementary material for: Road mitigation structures designed for Texas ocelots: Influence of structural characteristics and environmental factors on non-target wildlife usage
Source: PLoS One. 2024 Jul 22;19(7):e0304857. doi: 10.1371/journal.pone.0304857 (PMC11262682; doi:10.1371/journal.pone.0304857)
Supplement: S1 Table — All structures were monitored using remote cameras, and data were collected at WCS from January 2017 to May 2019. Openness ratio was calculated as width × height / length. (DOCX) [file pone.0304857.s005.docx]

Supplementary Table 1. Type and dimensions (width, height, and length in meters) of wildlife crossing structures (WCS) constructed by the Texas Department of Transportation to protect ocelots and other animals’ mortality along State Highway 100 in Cameron County, Texas, USA. All structures were monitored using remote cameras, and data were collected at WCS from January 2017 to May 2019. Openness ratio was calculated as width × height / length.

| Attribute | WCS1 | WCS2 | WCS3 | WCS3A | WCS4 |
| --- | --- | --- | --- | --- | --- |
| Underpass type | box culvert | box culvert | bridge | box culvert | box culvert |
| Dimensions | 3.0 x 2.1 x 48.8 | 3.0 x 2.1 x 54.9 | 6.1 x 2.0 x 22.6 | 1.8 x 1.2 x 35.1 | 3.0 x 1.5 x 24.4 |
| Openness ratio | 0.13 | 0.11 | 0.54 | 0.06 | 0.18 |
