# Supplementary material for: Road mitigation structures designed for Texas ocelots: Influence of structural characteristics and environmental factors on non-target wildlife usage
Source: PLoS One. 2024 Jul 22;19(7):e0304857. doi: 10.1371/journal.pone.0304857 (PMC11262682; doi:10.1371/journal.pone.0304857)
Supplement: S2 Table — Structures were constructed to protect ocelots and other animals’ mortality on State Highway 100 in Cameron County, Texas, between January 2017 and May 2019. (DOCX) [file pone.0304857.s006.docx]

Supplementary Table 2. List of species and species group recorded interacting with wildlife crossing structures and wildlife guards constructed by the Texas Department of Transportation. Structures were constructed to protect ocelots and other animals’ mortality on State Highway 100 in Cameron County, Texas, between January 2017 and May 2019.

| Common name | Scientific name |
| --- | --- |
| Virginia opossum | *Didelphis virginianus* |
| Nine-banded armadillo | *Dasypus novemcinctus* |
| American beaver | *Castor canadensis* |
| Nutria | *Myocastor coypus* |
| Mexican ground squirrel | *Ictidomys mexicanus* |
| Eastern cottontail | *Sylvilagus floridanus* |
| Black-tailed jackrabbit | *Lepus californicus* |
| White-tailed deer | *Odocoileus virginianus* |
| Javelina | *Tayassu tajacu* |
| Feral hog | *Sus scrofa* |
| Domestic cattle | *Bos taurus* |
| Nilgai | *Boselaphus tragocamelus* |
| Domestic sheep | *Ovis aries* |
| Bobcat | *Lynx rufus* |
| Ocelot | *Leopardus pardalis* |
| Domestic cat | *Felis catus* |
| Coyote | *Canis latrans* |
| Domestic dog | *Canis lupus familiarus* |
| Northern raccoon | *Procyon lotor* |
| Striped skunk | *Mephitis mephitis* |
| Long-tailed weasel | *Mustela frenata* |
| Texas tortoise | *Gopherus berlandieri* |
| Texas indigo snake | *Drymarchon melanurus erebennus* |
| Grouped herpetofauna |  |
