# Supplementary material for: Road mitigation structures designed for Texas ocelots: Influence of structural characteristics and environmental factors on non-target wildlife usage
Source: PLoS One. 2024 Jul 22;19(7):e0304857. doi: 10.1371/journal.pone.0304857 (PMC11262682; doi:10.1371/journal.pone.0304857)
Supplement: S3 Table — (DOCX) [file pone.0304857.s007.docx]

Supplementary Table 3. Descriptive statistics, including mean, standard deviation, and range, of quantitative factors being tested in the generalized linear model for data collected from May 2018 to May 2019 on State Highway 100 in Cameron County, Texas, USA.

|  | Mean ± SD | Range |
| --- | --- | --- |
| Daily Low Temperature (°C) | 18.4±6.8 | 2.2-27.2 |
| Openness ratio | 0.22±0.19 | 0.06-0.54 |
| WCS Height (m) | 1.63±0.37 | 1.2-2.1 |
| WCS Length (m) | 32.7±11.0 | 22.6-54.9 |
| Precipitation (cm) | 0.16±0.78 | 0.0-11.9 |
| Distance to Vegetation (m) | 12.4±17.7 | 0.0-83.9 |
