# Supplementary material for: Road mitigation structures designed for Texas ocelots: Influence of structural characteristics and environmental factors on non-target wildlife usage
Source: PLoS One. 2024 Jul 22;19(7):e0304857. doi: 10.1371/journal.pone.0304857 (PMC11262682; doi:10.1371/journal.pone.0304857)
Supplement: S4 Table — Average number of individuals denotes average number of individuals crossing per month, and percent (%) contributed refers to the extent to which each species contributed to the clustering of the species observed crossing within each group. (DOCX) [file pone.0304857.s008.docx]

Supplementary Table 4. Most abundant species crossing at wildlife crossing structure (WCS) 3 and WCS4 post construction, based on similarity percentage analysis of data collected from May 2018 to May 2019 in Cameron County, Texas. Average number of individuals denotes average number of individuals crossing per month, and percent (%) contributed refers to the extent to which each species contributed to the clustering of the species observed crossing within each group.

| Site | Post | | |  |
| --- | --- | --- | --- | --- |
|  | Species | Average no. individuals | % Contributed | |
| WCS3 | Opossum | 20.9 | 77.7 | |
|  | White-tailed deer | 2.0 | 11.3 | |
|  | Raccoon | 1.3 | 5.5 | |
|  | Eastern cottontail | 2.3 | 3.9 | |
|  | Nine-banded armadillo | 0.5 | 0.7 | |
|  | Bobcat | 0.9 | 0.4 | |
|  | Northern bobwhite | 1.1 | 0.4 | |
|  | Striped skunk | 0.2 | 0.1 | |
| WCS4 | Striped skunk | 29.2 | 45.1 | |
|  | Eastern cottontail | 42.6 | 29.1 | |
|  | Opossum | 32.6 | 18.5 | |
|  | Coyote | 2.4 | 4.1 | |
|  | Raccoon | 1.8 | 3.2 | |
|  | Long-tailed weasel | 0.4 | 0.1 | |
